# Emergent structural and functional properties of hippocampal multi-cellular aggregates

**DOI:** 10.3389/fnins.2023.1171115

**Published:** 2023-06-15

**Authors:** Victor P. Acero, Suradip Das, Olivia Rivellini, Erin M. Purvis, Dayo O. Adewole, Daniel Kacy Cullen

**Affiliations:** ^1^Center for Brain Injury and Repair, Department of Neurosurgery, Perelman School of Medicine University of Pennsylvania, Philadelphia, PA, United States; ^2^Center for Neurotrauma, Neurodegeneration and Restoration, Corporal Michael J. Crescenz Veterans Affairs Medical Center, Philadelphia, PA, United States; ^3^Department of Bioengineering, School of Engineering and Applied Science, University of Pennsylvania, Philadelphia, PA, United States; ^4^Department of Neuroscience, Perelman School of Medicine, University of Pennsylvania, Philadelphia, PA, United States

**Keywords:** *In vitro* hippocampal culture, 3D neuron cultures, astrocyte domains, neuronal polarization, *in vitro* electrophysiology, neural spheroid

## Abstract

Hippocampal neural networks are distinctly capable of integrating multi-modal sensory inputs to drive memory formation. Neuroscientific investigations using simplified *in vitro* models have greatly relied on planar (2D) neuronal cultures made from dissociated tissue. While these models have served as simple, cost-effective, and high-throughput tools for examining various morphological and electrophysiological characteristics of hippocampal networks, 2D cultures fail to reconstitute critical elements of the brain microenvironment that may be necessary for the emergence of sophisticated integrative network properties. To address this, we utilized a forced aggregation technique to generate high-density (>100,000 cells/mm^3^) multi-cellular three-dimensional aggregates using rodent embryonic hippocampal tissue. We contrasted the emergent structural and functional properties of aggregated (3D) and dissociated (2D) cultures over 28 days *in vitro* (DIV). Hippocampal aggregates displayed robust axonal fasciculation across large distances and significant neuronal polarization, i.e., spatial segregation of dendrites and axons, at earlier time points compared to dissociated cultures. Moreover, we found that astrocytes in aggregate cultures self-organized into non-overlapping quasi-domains and developed highly stellate morphologies resembling astrocyte structures *in vivo*. We maintained cultures on multi-electrode arrays (MEAs) to assess spontaneous electrophysiological activity for up to 28 DIV. We found that 3D networks of aggregated cultures developed highly synchronized networks and with high burstiness by 28 DIV. We also demonstrated that dual-aggregate networks became active by 7 DIV, in contrast to single-aggregate networks which became active and developed synchronous bursting activity with repeating motifs by 14 DIV. Taken together, our findings demonstrate that the high-density, multi-cellular, 3D microenvironment of hippocampal aggregates supports the recapitulation of emergent biofidelic morphological and functional properties. Our findings suggest that neural aggregates may be used as segregated, modular building blocks for the development of complex, multi-nodal neural network topologies.

## 1. Introduction

Hippocampal neural networks facilitate multisensory integration of experience into distinct memories, as well as memory retrieval (Pfeiffer and Foster, 2015; Sasaki et al., 2018; Frank and Wiley, 2019). Pathological modifications in these networks, e.g., Alzheimer’s or traumatic brain injury (TBI), impair healthy learning and memory (Dere et al., 2010; Wolf et al., 2017). Advancements in our understanding of these networks will improve our fundamental knowledge of memory processes and our capacity to clinically address hippocampal network pathologies. Invasive, e.g., implanted electrodes, and non-invasive, e.g., fMRI, experimental techniques have been used to study rodent, feline, porcine, non-human primate, and human hippocampal networks during behaviorally relevant activities (e.g., memory tasks and spatial navigation) and have advanced our capacity to reduce complex behavior to fundamental neural activity (Castellanos and Proal, 2012; Smith et al., 2012; Paterno et al., 2017; Ulyanova et al., 2018; Frank and Wiley, 2019). Despite the advances in methodology and animal models, our understanding of how hippocampal networks support their unique functionality remains obscured by the biological complexity of the underlying processes. There have been notable success with *ex vivo* brain slice cultures which can preserve elements of *in vivo* morphology, cytoarchitecture, and anatomical connectivity; however, slice cultures are still relatively complex systems and the process of generating slice cultures is morphologically damaging (Yang et al., 2014; Gulino et al., 2019). Thus, researchers often turn to *in vitro* preparations – which offer a simplified system with greater degree of experimental control and spatiotemporal resolution – to advance our understanding of hippocampal network function. Here, the standard experimental approach uses dissociated neuronal tissue to generate two-dimensional (2D) network models, typically of randomly organized neural networks on multi-electrode arrays (MEA), which are simple, reproducible, low cost, high throughput tools that have aided in disease modeling and drug exploration for decades. However, 2D preparations do not accurately reproduce crucial aspects of the *in vivo* microenvironment, namely, appropriate densities (>100,000 cells/mm^3^), the cytoarchitecture of astrocyte-neuron interactions, and multi-dimensionality (3D). Indeed, planar models poorly recapitulate emergent properties inherent to *in vivo* neuronal networks – including hippocampal networks – such as complex patterns of rhythmic activity in characteristic frequency domains.

These modeling challenges may be partially due to characteristic cell and network level features that have been vexing for researchers to recapitulate, for instance, axonal fasciculation, neuronal polarization, astrocyte arborization, astrocyte domain formation. Axonal fasciculation is the process by which path seeking or post-synaptic axons adhere together forming a tight rope-like bundle known as a fascicle. Fasciculation is necessary *in vivo* for the establishment of proper neuronal connections over long distances with their target site (Barry et al., 2010). Although fascicle formation can be stimulated *in vitro* with dissociated neuronal cultures, they are relatively thin (~5 μm) and defasciculate after traversing short distances (Wakade et al., 2013; Šmít et al., 2017). Fasciculation may also drive neuronal polarization *in vivo* and *in vitro* via triggering of mechanical and biochemical signaling (Barry et al., 2010). Neuronal polarization, i.e., the functional and structural asymmetry arising from distinct dendritic and axonal compartments, is ubiquitous throughout the brain and necessary for the function of neuronal networks. Dissociated self-organized neuronal cultures often demonstrate overlap of both axon and dendrite associated proteins (Tuj1 and MAP2, respectively) within single neurites, and a lack of clear spatial segregation of dendrites and axons in the network, unlike neurons and axonal tracts *in vivo*.

Astrocytes are highly arborized glial cells which self-organize into tessellating unique spatial domains. In this arrangement, morphologically complex astrocytes can bidirectionally sense and engage with thousands of synapses (via tripartite synapse formations) and communicate within astrocytic networks via gap-junctions, thus uniquely regulating neural network processes and functional outputs of the central nervous system. Astrocytes have been shown to modulate synchronization, bursting parameters, and morphological maturation in hippocampal co-cultures (Feldt et al., 2010). The use of astrocyte conditioned media, rich in beneficial growth factors, is generally sufficient for enhancing morphological and functional maturation of neuronal cultures (Feldt et al., 2010; Pozzi et al., 2017; Teneggi et al., 2021). However, attempts to recapitulate the arborized morphology of astrocytes *in vitro* have had limited success, because they are often cultured in media supplemented with fetal bovine serum (FBS) in order to promote survival and proliferation. FBS has high levels of lipopolysaccharides and extracellular vesicles found to alter cellular phenotype expression. Hence, astrocytes cultured in serum containing media exhibit reactive phenotypes with round and low-process morphology and abnormally high GFAP expression, as opposed to a more arborized, stellate, quiescent, domain-forming phenotypes seen *in vivo*. To date, astrocyte domains remain exclusively an *in vivo* phenomenon mediated by highly intricate biochemical cues in the local microenvironment (Freeman, 2010) and are yet to be recapitulated under *in vitro* culture conditions. The attenuation and/or absence of these fundamental morphological processes *in vitro* highlights some of the challenges toward developing a biofidelic microenvironment.

The diverse array of emergent functionality found in brain networks is facilitated by complex spatiotemporal patterns of coordinated, persistent, and rhythmic activity of neuronal ensembles, e.g., theta frequency (4–10 Hz) for place cell activation. In the hippocampus, high-frequency ripple (100–200 Hz) oscillations appear to drive memory consolidation in the hippocampus and neocortex (Butler and Paulsen, 2015; Jahnke et al., 2015). Additionally, hippocampal networks (*in vivo* and *in vitro*) demonstrate greater reliance on the bursting activity of neuronal ensembles to drive functionality and manipulate information. Coordinated oscillations in the theta frequency band are associated with learning during memory tasks or spatial navigation. Likewise, increased theta-gamma (30–80 Hz) phase-amplitude coupling is associated with successful memory encoding (Onslow et al., 2011; Butler and Paulsen, 2015). Notably, machine learning classifiers could use theta bursting and burstiness metrics to distinguish between hippocampal and cortical networks *in vitro* (Charlesworth et al., 2015). The translatability of *in vitro* models of neural networks can thus be measured against the electrophysiological characteristics and their similarity to their corresponding circuits *in vivo*.

We assert that there are generally key limitations of conventional 2D cell culture techniques that limit their ability to recapitulate the above discussed *biofidelic* structural and functional properties typical of hippocampal networks *in vivo*. To address these limitations, our group has utilized our extensive experience in generating high-density 3D (>100,000 cells/mm^3^) populations of cells using a forced aggregation technique to create hippocampal aggregates that can act as nodes in tissue engineered models of neural networks (Struzyna et al., 2015, 2017; Dhobale et al., 2018; Cullen et al., 2019; Marinov et al., 2020; Adewole et al., 2021). Our 3D, high-density, multi-cellular, aggregates may recapitulate the necessary microenvironment for appropriate cellular and whole-tissue maturation. Similarly, we expect that aggregates will drive the development of more phenotypically appropriate spontaneous activity in the aggregate network.

**Figure 1:**
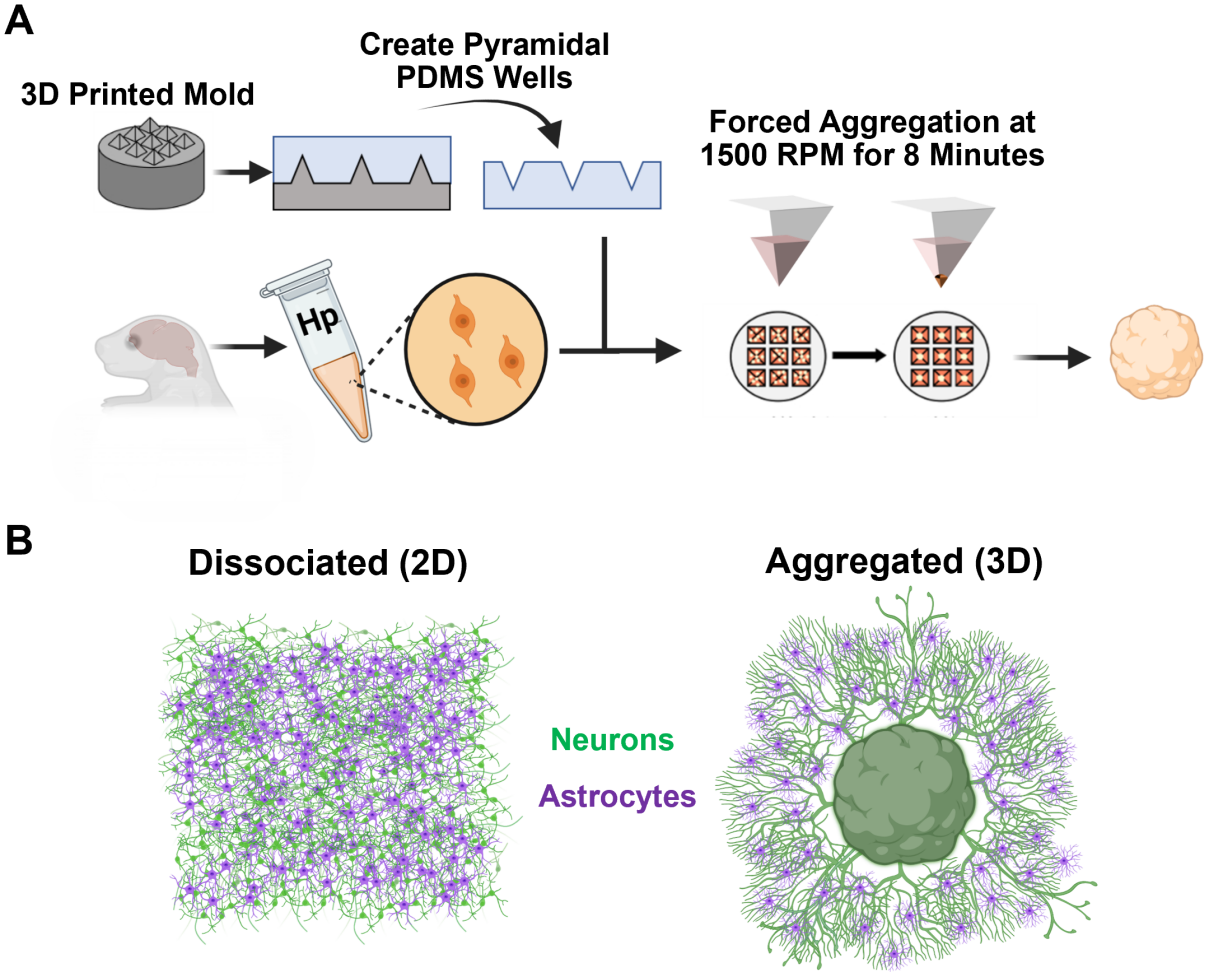
Experimental approach. **(A)** We utilized 3D printed molds to generate inverted pyramidal PDMS wells. Rat E18 dissociated neuronal suspension was transferred to the inverted pyramidal PDMS wells (50,000 cells/well), centrifuged at 1500 rpm for 8 min, and incubated overnight to allow the newly formed aggregate to stabilize. **(B)** This study examined the emergent morphological and functional differences between dissociated (2D) and aggregated (3D) hippocampal neuron-astrocyte cultures.

In the present study, we compare the morphological properties of 2D dissociated and 3D aggregated cultures of hippocampal cells to investigate the effect of three-dimensionality on neuronal-glial development (Figure 1). Further, we also report unique electrophysiological characteristics exhibited by 3D hippocampal aggregates and their potential application as nodes in simulating neural networks.

## 2. Materials and methods

### 2.1. Hippocampal tissue isolation and culture of dissociated cells

All procedures involving animals were approved by the Institutional Animal Care and Use Committee of the University of Pennsylvania and followed the National Institutes of Health Guide for the Care and Use of Laboratory Animals (NIH Publications No. 80–23; revised 2011).

Sprague Dawley rats (Charles River) were the primary source of embryonic day 18 (E18) hippocampal neurons. Individual hippocampi were dissected and enzymatically dissociated in prewarmed trypsin (0.25%) + EDTA (1 mM) at 37°C for 15 min. Following the trypsin neutralization with DMEM +10% FBS, the solution was replaced with deoxyribonuclease (DNase) (0.15 mg/mL) in neuronal culture media composed of Neurobasal + B27 + Glutamax (Thermo Fisher Scientific). Dissociated tissue + DNase was centrifuged for 10 min at 300 G before DNase was removed and the cells were mechanically triturated and resuspended in neuronal culture media. Dissociated hippocampal cells were plated in planar culture at a density of ~25,000 cells/cm^2^. All cell culture surfaces were pre-treated with poly-L-lysine (PLL, Sigma, 0.01% w/v) or polyethylenimine (PEI, Sigma, 0.1% w/v) in 1x borate buffer solution overnight followed by pre-treatment with laminin for 2 h. Cultures were maintained using neuronal media, and 2/3 of media was replaced every 2–3 days *in vitro* (DIV). No glial inhibitors were added, because of the well documented positive effects of astrocytes on survival and maturation of hippocampal cultures (Feldt et al., 2010; Pozzi et al., 2017). Cultures were plated for quantitative analysis of astrocyte morphology (2D: DIV 7, *n* = 6; DIV 14, *n* = 4; DIV 21, *n* = 4; DIV 28, *n* = 4) and axon-dendrite colocalization (2D: DIV 7, *n* = 5; DIV 14, *n* = 5; DIV 21, *n* = 5; DIV 28, *n* = 5). Additionally, cultures were plated for qualitative morphological assessments and characterization (2D: DIV 7, *n* = 2; DIV 14, *n* = 2; DIV 21, *n* = 2; DIV 28, *n* = 2).

We employed microdissection tools to segment intact embryonic hippocampi into smaller explants. The segmentation process was conducted visually, utilizing operator judgment for size determination. Using our established protocol for dissociated hippocampal cultures, we maintained these segmented explants up to 7 days *in vitro* (DIV).

### 2.2. Hippocampal aggregate generation and culture

To create cell aggregates, dissociated hippocampal tissue was suspended at a density of 3–3.5 million cells/ml and transferred to an array of inverted pyramidal wells made in polydimethylsiloxane (PDMS) (Sylgard 184, Dow Corning) cast from a custom-designed, 3D printed mold (Figure 1A). Cell suspensions were then centrifuged in the wells at 1500 rpm for 8 min; this centrifugation resulted in forced aggregation of neurons with precise control of the number of neurons per aggregate/sphere (~50,000 cells per aggregate; 15 μL of cell suspension per well) (Figure 1A). Aggregates were incubated in the inverted pyramidal wells overnight at 37°C, 5% CO2 to allow cell–cell adhesions to structurally reinforce the aggregate. After incubation for 24–48 h, aggregates were easily removed from pyramidal wells using a 200 μL pipette tip and then placed on the cell culture surface. Pyramidal well designs and forced aggregation protocols were previously validated using various cell/tissue sources (Ungrin et al., 2008; Struzyna et al., 2017; Serruya et al., 2018; Struzyna et al., 2018; Katiyar et al., 2019; Adewole et al., 2021; Gordián-Vélez et al., 2021; Struzyna et al., 2022). Protocols for cell culture surface treatment, neuronal media composition, and media changes were identical to that of dissociated hippocampal cultures. Aggregate cultures were plated for quantitative analysis of astrocyte morphology (3D: DIV 7, *n* = 4; DIV 14, *n* = 4; DIV 21, *n* = 4; DIV 28, *n* = 4) and axon-dendrite colocalization (3D: DIV 7, *n* = 4; DIV 14, *n* = 6; DIV 21, *n* = 5; DIV 28, *n* = 6). Additionally, cultures were plated for qualitative morphological assessments and characterization (3D: DIV 7, *n* = 2; DIV 14, *n* = 2; DIV 21, *n* = 2; DIV 28, *n* = 4).

### 2.3. Phase contrast microscopy and immunofluorescence confocal microscopy

Phase-contrast microscopy images of hippocampal cultures were taken at 10x magnification using a Nikon Eclipse Ti-S microscope, paired with a QIClick camera, and NIS Elements BR 4.13.00. Phase images were used to monitor cultures across experimental timepoints to assess culture health and growth before terminal experiments. Hippocampal aggregates were opaque due to the thickness and density of the microtissue, so light microscopy only revealed structures outside and at the edges the aggregates.

To characterize neuronal and astrocyte morphological maturation, dissociated and aggregated hippocampal cultures were fixed in 4% formaldehyde for 35 min at 7, 14, 21, and 28 DIV. Cultures were rinsed in 1x PBS and permeabilized with 0.3% Triton X-100 + 4% horse serum in PBS for 60 min before being incubated with primary antibodies overnight at 4°C. Primary antibodies were Tuj-1/β-tubulin III (1:500; T8578, Sigma-Aldrich) to label axons, microtubule associated protein-2 (MAP2; 1:10000; AB5392, ABCAM) to label neuronal somata and dendrites, and glial-fibrillary associated protein (GFAP; 1:500; AB53554, ABCAM) to label astrocytes. Following primary antibody incubation, cultures were rinsed with 1x PBS and incubated with fluorescently labeled secondary antibodies (Alexa Fluor; 1:500) for 2 h at room temperature. Last, Hoechst (1:10,000; 33,342, Thermo Fisher Scientific) was added for 10 min at room temperature before rinsing in 1x PBS. Cultures were imaged on a Nikon A1RSI laser scanning confocal microscope paired with NIS Elements AR 4.50.00. All samples were imaged with a 10x objective and 1.5x digital zoom.

Scanning electron microscopy experiments were carried out at CDB Microscopy Core (Perelman School of Medicine, University of Pennsylvania). Samples were washed three times with 50 mM Na-cacodylate buffer, fixed for 2 h with 2% glutaraldehyde in 50 mM Na-cacodylate buffer (pH 7.3), and dehydrated in a graded series of ethanol concentrations through 100% over a period of 2.5 h. Dehydration in 100% ethanol was done three times. After the 100% ethanol step, dehydrated samples were incubated for 20 min in 50% HMDS in ethanol followed by three changes of 100% HMDS (Sigma-Aldrich) and followed by overnight air-drying as described previously (Braet et al., 1997). Then samples were mounted on stubs and sputter coated with gold palladium. Specimens were observed and photographed using a Quanta 250 FEG scanning electron microscope (FEI, Hillsboro, OR, United States) at 10 kV accelerating voltage.

### 2.4. Quantification of neuronal polarization

The evolution of axonal-dendritic compartmentalization was examined using Tuj1:MAP2 double immunolabeled micrographs acquired using confocal microscopy. A 25-pixel diameter rolling ball background subtraction function in ImageJ was used to prepare images for colocalization analysis. The EZColocalization ImageJ plugin developed by Stauffer et al. (2018) was used to quantify the spatial overlap of Tuj1+ and MAP2+ neurites. All pixels were sampled to calculate the Pearson Correlation Coefficient. The top 10% of pixels (based on intensity) were sampled to calculate the Threshold Overlap Score (Stauffer et al., 2018). The neuronal polarization between culture conditions across time was quantified utilizing the Mander’s Overlap Coefficients (MOC) M1 and M2 as measures of Tuj1 and MAP2 co-localization. M1 is the area ratio of signal A (Tuj1) pixels co-occurring with signal B (MAP2), to the total number of pixels for signal A (Tuj1). Likewise, M2 is an area ratio of signal B (MAP2) pixels co-occurring with signal A (Tuj1) pixels to the total number of pixels for signal B (MAP2). We used a Sidak’s multiple comparisons test to examine significance, because each data point (M1 and M2 at a given DIV) is independently measured. We used two-way ANOVA multiple comparisons tests to assess statistical significance and corrected for multiple comparisons using the Holm-Sidak method. We considered significance at *p*-values lower than 0.05.

### 2.5. Quantification of astrocyte morphology

Specific features related to astrocyte morphology and process arborization were examined using GFAP+ immunofluorescence micrographs acquired using confocal microscopy. Two image analysis protocols were used to characterize the morphological maturation of hippocampal astrocytes. All analyses were conducted using ImageJ. Astrocyte processes were hand-traced with the Simple Neurite Tracer (SNT) tool in ImageJ. Sholl analysis about the center of each soma was applied to automatically retrieve quantitative descriptors for statistical comparisons related to the extent of astrocyte arborization (see Supplementary Figure 1, for example). We used the SNT tool to extract morphometric data from 10 astrocytes per culture. We used non-parametric Kolmogorov–Smirnov tests and corrected for multiple comparisons using the Holm-Sidak method to assess significant differences in the distribution of values at various distances. We considered significance at *p*-values lower than 0.05.

Additionally, we quantified several morphometric features for each sampled astrocyte, including the number and length of main branches, number of junctions per branch, and total length of all processes. Main branches were identified as processes originating directly from the soma and were measured from the base of the soma to the longest terminal on its branch. Notably small projections from the soma bearing no junctions were not considered as main branches. Junctions (branching processes from main branches) were totaled per branch and measured to calculate the total process length for each astrocyte. Projections shorter than 5 μm were not considered individual junctions. We analyzed the morphological parameters of astrocytes using two complementary methods: cell-based and culture-based. The cell-based approach analyzed the values of each individual astrocyte, while the culture-based approach averaged the values of all the astrocytes in a given culture. We used two-way ANOVA multiple comparisons tests to assess statistical significance and corrected for multiple comparisons using the Holm-Sidak method. We considered significance at *p*-values lower than 0.05.

We quantified the astrocyte density (cells/mm^2^) in dissociated and aggregate cultures. In the aggregate cultures, we divided the analysis into three ‘Zones’: Zone 1 (50–200 μm), Zone 2 (200–400 μm), and Zone 3 (400–600 μm) from the aggregate perimeter (Supplementary Figure 4). We used one-way ANOVA to assess statistical significance and corrected for multiple comparisons using Dunn’s method. We considered significance at *p*-values lower than 0.05.

### 2.6. Recording spontaneous activity with multi-electrode arrays

All multi-electrode array (MEA) surfaces were pre-treated with 0.1% polyethylenimine (PEI) in 1X borate buffer solution overnight followed by pre-treatment with laminin for 2 h. We utilized two distinct *in vitro* MEA and acquisition systems in the study. We cultured single-aggregate and dissociated hippocampal cultures on Axion Biosystems 6-well MEA plates where each well contained an array of 64 planar PEDOT electrodes arranged in a 8 × 8 grid with 7 grounding electrodes. The electrical activity of cultures was recorded using the Maestro Edge MEA Acquisition System (Axion Biosystem) at a sampling frequency of 12.5 kHz using AxIS acquisition software. Raw voltage signals were bandpass filtered from 200 to 3,000 Hz and spikes were detected by thresholding to 6 times the standard deviations (SD) from the root mean square (RMS) of the signal. Recordings were conducted for 10 min in a 37°C heated chamber.

We cultured dual-aggregate systems in MED-64 MEA dishes that contained 64 planar platinum black electrodes arranged in an 8 × 8 grid, with 4 larger reference electrodes that served as ground nodes. Two aggregates were placed diagonally on separate corners of the MEA. The activity of all cultures was recorded using an MEA recording adapter frame (AL-MED-C03, AutoMate Scientific) to which two 32-channel digital headstages (Omnetics) were connected. After pre-amplification by the headstages, the 64 recording channels were fed into a neural data acquisition system (Digital Lynx, Neuralynx) connected to a computer running the acquisition software suite (Cheetah, Neuralynx) that manages the acquisition system. During each experimental session, continuous voltage signals were recorded from each MEA electrode, amplified, and sampled at 32 kHz with a 24-bit resolution. Raw voltage signal bandpass filtered and spikes were detected when the voltage exceeded 6 SD from the RMS of the signal. We used spike times to calculate firing rates and to generate raster plots of activity across electrodes.

### 2.7. Analyzing spontaneous activity

All electrophysiology data preparation and analysis was conducted on MATLAB version R2022b. We calculated the mean interspike interval (ISI) by measuring the time difference between each spike event and excluding any values greater than 100 ms. The mean firing frequency (Hz) was derived from the inverse of the ISI. We modified a previously reported burst detection MATLAB script to detect bursts and extract useful parameters, such as burst duration, interburst interval, firing frequency, and number of spikes (Morozova et al., 2020). We chose to define bursts as discrete events containing at least 5 spikes, with a minimum ISI of 100 ms to initiate a burst, and a maximum ISI of 250 ms to continue a burst, because similar thresholds have been previously reported for *in vitro* hippocampal cultures (Charlesworth et al., 2015; Tedesco et al., 2018). We considered an electrode to be “active” if had >1 spike throughout the length of the recording. We used two-way ANOVA multiple comparisons tests to assess statistical significance and corrected for multiple comparisons using the Holm-Sidak method. We performed these tests in two ways: within each group across timepoints and between culture conditions at each timepoint. We assessed significance using the mean of each quantified metric across active electrodes and did not test for significance using individual electrode values. Individual electrode values are shown for relevant metrics to provide additional context on within-group variability for cultures. We considered significance at *p*-values lower than 0.05.

## 3. Results

### 3.1. Neurons in high-density three-dimensional aggregate cultures show robust axonal fasciculation

**Figure 2:**
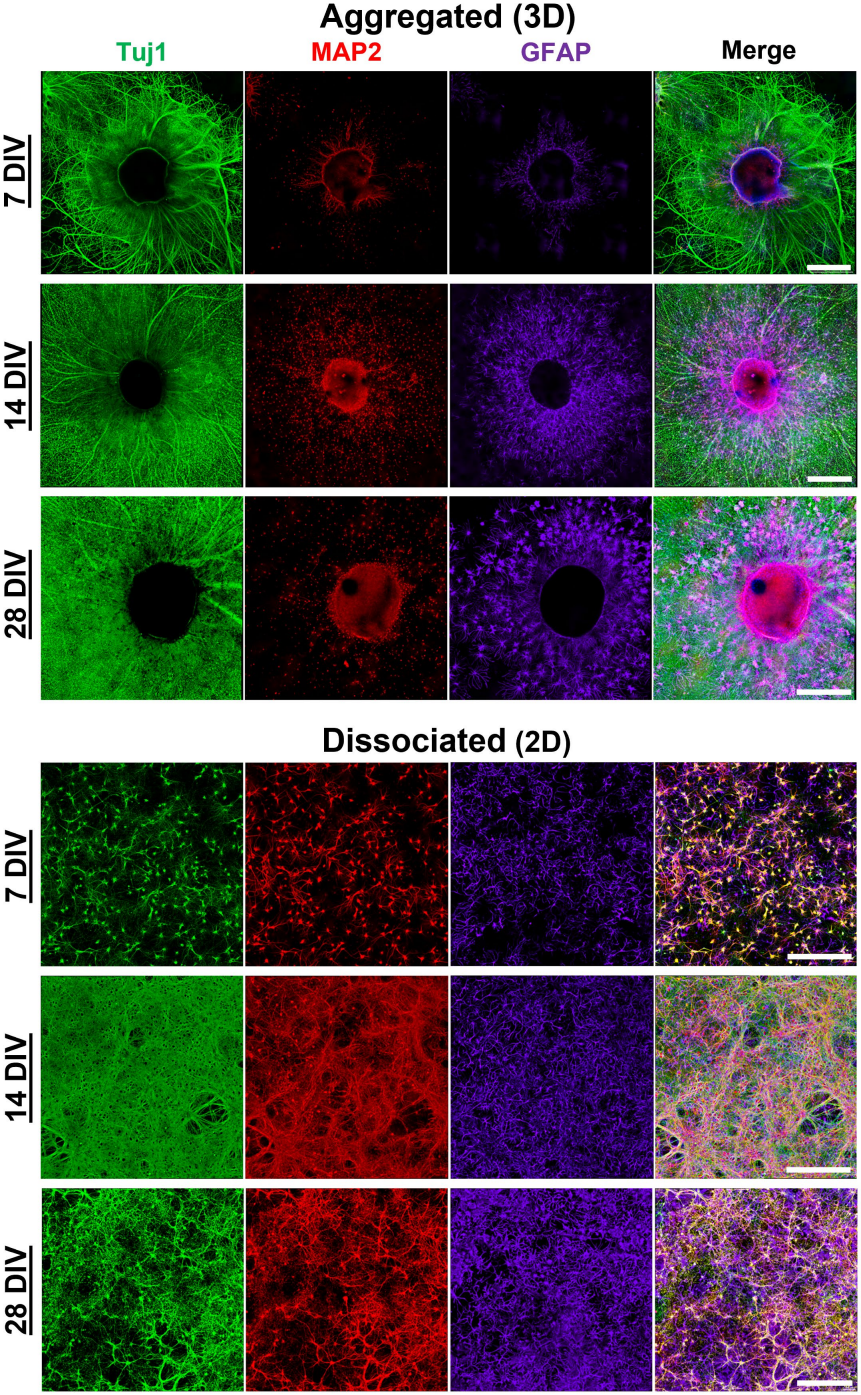
Morphological maturation of aggregated and dissociated hippocampal cultures. Morphological maturation of aggregated and dissociated hippocampal cultures, specifically axon (Tuj1), dendrites (MAP2), and astrocytes (GFAP) was visualized utilizing immunofluorescence confocal microscopy. Hippocampal aggregates demonstrate robust fasciculation of axonal projections which project 500–1000 μm from the edge of the aggregate as early as 7 DIV. Moreover, we found that MAP2+ dendrite growth was predominantly restricted to the most proximal (<50 μm) area relative to the aggregate body. Astrocytes were shown to survive in both culture conditions. In aggregated cultures, astrocytes appeared to demonstrate greater arborization relative to dissociated cultures. Astrocytes in aggregated culture appears to self-organize into domain-like structures, rather than clustering as was seen in dissociated cultures. Scale bar = 500 μm.

**Figure 3:**
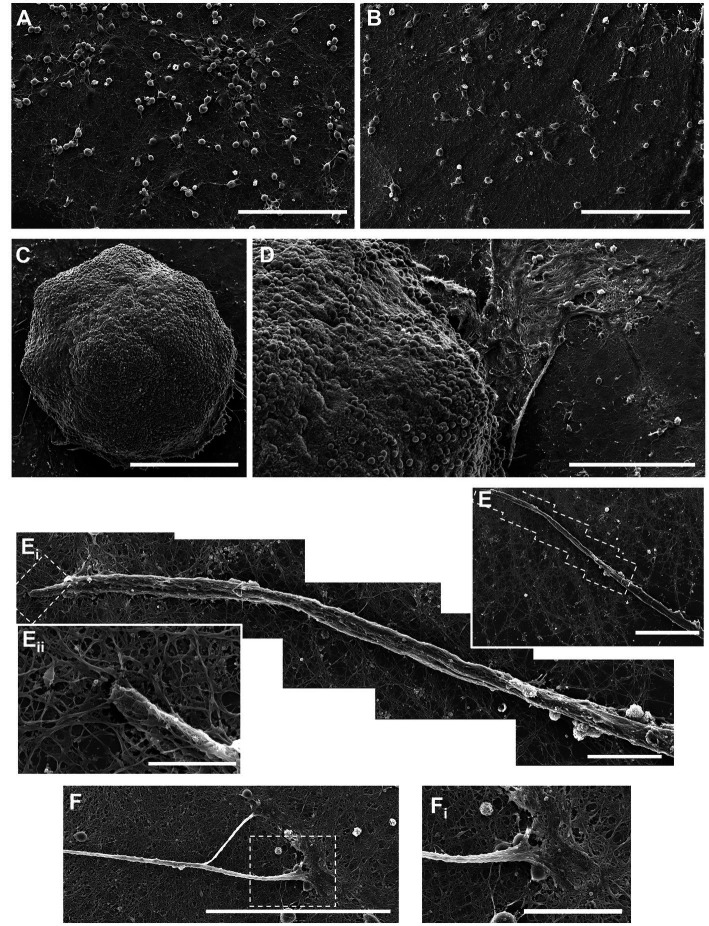
Scanning electron microscope (SEM) images of aggregated and dissociated hippocampal cultures. **(A,B)** SEM images of dissociated hippocampal cultures growing on planar surfaces. **(C)** SEM image of the aggregate body shows the high-density three-dimensional packing of neuronal somata and self-generated extracellular matrix that likely supports aggregate cohesion. **(D)** Processes protrude from the aggregate body onto the planar cell culture surface. **(E)** Thick bundles of axons emerge due to the fasciculation of numerous individual neurite processes. **(F)** Axon fascicles also spontaneously defasciculate and merge with the surround bed of neurites on the planar surface. **(A,B)** Scale bar = 100 μm; **(C,D)** scale bar = 250 μm; **E**_i_ to **E**, **E**_ii_ to **E**_i_ and **E**_iii_ to **E**_ii_; **(F)** scale bar = 100 μm; **(F**_i_**)** scale bar = 30 um.

We utilized a forced aggregation method to generate multi-cellular hippocampal aggregates (Figure 1A). We assessed morphological differences between dissociated (2D) and aggregated (3D) cultures containing both astrocytes and neurons (Figure 1B). Our initial characterization used immunocytochemistry and scanning electron microscopy (SEM) to qualitatively assess differences in neurite growth over time. We found that both culture conditions demonstrated healthy neurite extension across all time points assessed. By 7 DIV, neurons in 2D cultures formed a fine mesh of processes (Figures 2, 3A,B). These neurons in 2D culture tended to undergo progressive clustering as neurites pulled together spatially proximal soma (Figure 2). At later time points in 2D cultures, parallel neurites formed short-spanning bundles between small groupings of neurons. In contrast to dissociated cultures of planar neurons, aggregate cultures enable high-density (>100,000 cells/mm^3^) three-dimensional packing of neuronal somata and self-generated extracellular matrix that likely supported aggregate cohesion (Figure 3C). Hippocampal aggregates demonstrated robust fasciculation of axonal (Tuj1+) projections which extended 500–1,000 μm from the edge of the aggregate as early as 7 DIV (Figure 2). These fascicles could span long distances, merge with other fascicles, separate into thinner fascicules, and, at the distal end, would defasciculate into individual axonal process (Supplementary Figure 2). However, not all neurite outgrowth fasciculated into large bundles; there was also a surrounding area of dense mesh-like Tuj1+ neurite growth that increased in coverage from 7 to 28 DIV (Figures 2, 3D). While fascicles often emerged directly from the aggregate body, we also observed fascicles that formed spontaneously from a dense mesh of neurites (Figure 3E–E_ii_). We found examples of large axon fascicles defasciculating as well (Figure 3F, 3F_i_). Notably, these large fascicles were distinct to aggregate cultures, as well as whole tissue hippocampal explants (Supplementary Figure 5), and were observed as early as 7 DIV.

### 3.2. Hippocampal aggregate cultures exhibit neuronal polarization

**Figure 4:**
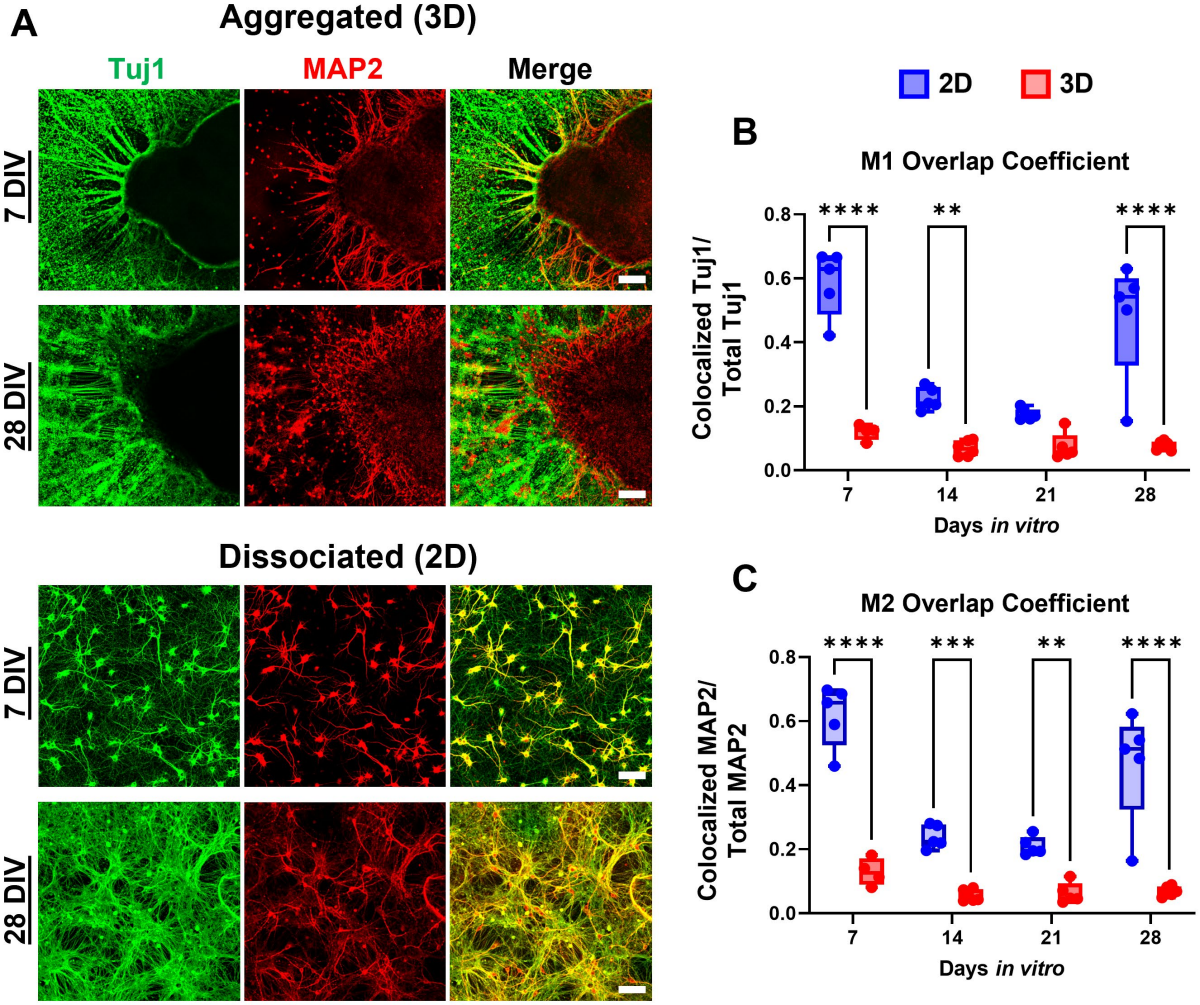
Aggregated neurons demonstrate reduced MAP2:Tuj1 colocalization, indicating increased polarity. **(A)** We assessed both the spatial overlap and co-labeling of neurites with Tuj1/MAP2 in aggregated versus dissociated cultures. **(B,C)** We quantified neuronal polarization between culture conditions across time utilizing the Mander’s Overlap Coefficients (MOC) M1 and M2 as measures of Tuj1 and MAP2 co-localization. Across timepoints there was significantly greater Tuj1-MAP2 colocalization in dissociated (2D) cultures relative to aggregated (3D) cultures, however the difference between culture conditions was most pronounced at earlier (7 and 14 DIV) time points. For M1: 7 DIV, *p* < 0.0001; 14 DIV, *p* = 0.0049; 28 DIV, *p* < 0.0001. For M2: 7 DIV, *p* < 0.0001; 14 DIV, *p* = 0.0006; 21 DIV, *p* = 0.0037; 28 DIV, *p* < 0.0001. Scale bar = 100 μm. The significance levels were denoted as follows: ** for *p* < 0.01, *** for *p* < 0.001, and **** for *p* < 0.0001.

Neuronal polarization refers to distinct spatial separation of dendritic and axonal processes. In the hippocampal aggregate cultures, MAP2+ dendrite growth was restricted to the most proximal (<50 μm) area to the centroid of the aggregate (see Figure 2). Qualitatively there was a near complete absence of MAP2/Tuj1 co-labeling within neurite compartments in aggregated cultures (Figure 4A). In contrast, dissociated cultures had a high degree of MAP2/Tuj1 co-labeling within neurite compartments (Figure 4A), which has been observed in previous studies (van Niekerk et al., 2022). M1 (Tuj1:MAP2 colocalization) values were significantly greater in dissociated (2D) cultures relative to aggregated (3D) cultures, and differences were most significant at 7 and 28 DIV (Figures 4B,C). Likewise, M2 (MAP2:Tuj1 colocalization) values in dissociated cultures were significantly higher relative to aggregated cultures at all timepoints, but differences were most significant at 7 and 28 DIV. Notably, dissociated cultures demonstrated significant decreases in M1 and M2 values at 14 and 21 DIV (For M1 and M2: 7 or 28 DIV to 14 or 21 DIV, *p* < 0.0001), but values were similar between 7 and 28 DIV. In contrast, aggregated cultures showed no significant differences in M1 and M2 values over time. Mixed effects analysis showed time and culture conditions independently as well as synergistically were significant sources of variation in both M1 and M2 coefficient scores. We also measured the Pearson’s Correlation Coefficient (PCC) and Threshold Overlap Score (TOS) as alternative ways of quantifying the MAP2/Tuj1 colocalization. We found similar changes in colocalization over time and significant differences between groups (Supplementary Figure 3). Altogether, the results demonstrate that hippocampal aggregates, but not dissociated cultures, exhibit early neuronal polarization which is maintained throughout maturation.

### 3.3. Astrocytes in aggregate cultures demonstrate domain-like self-organization and high arborization

**Figure 5:**
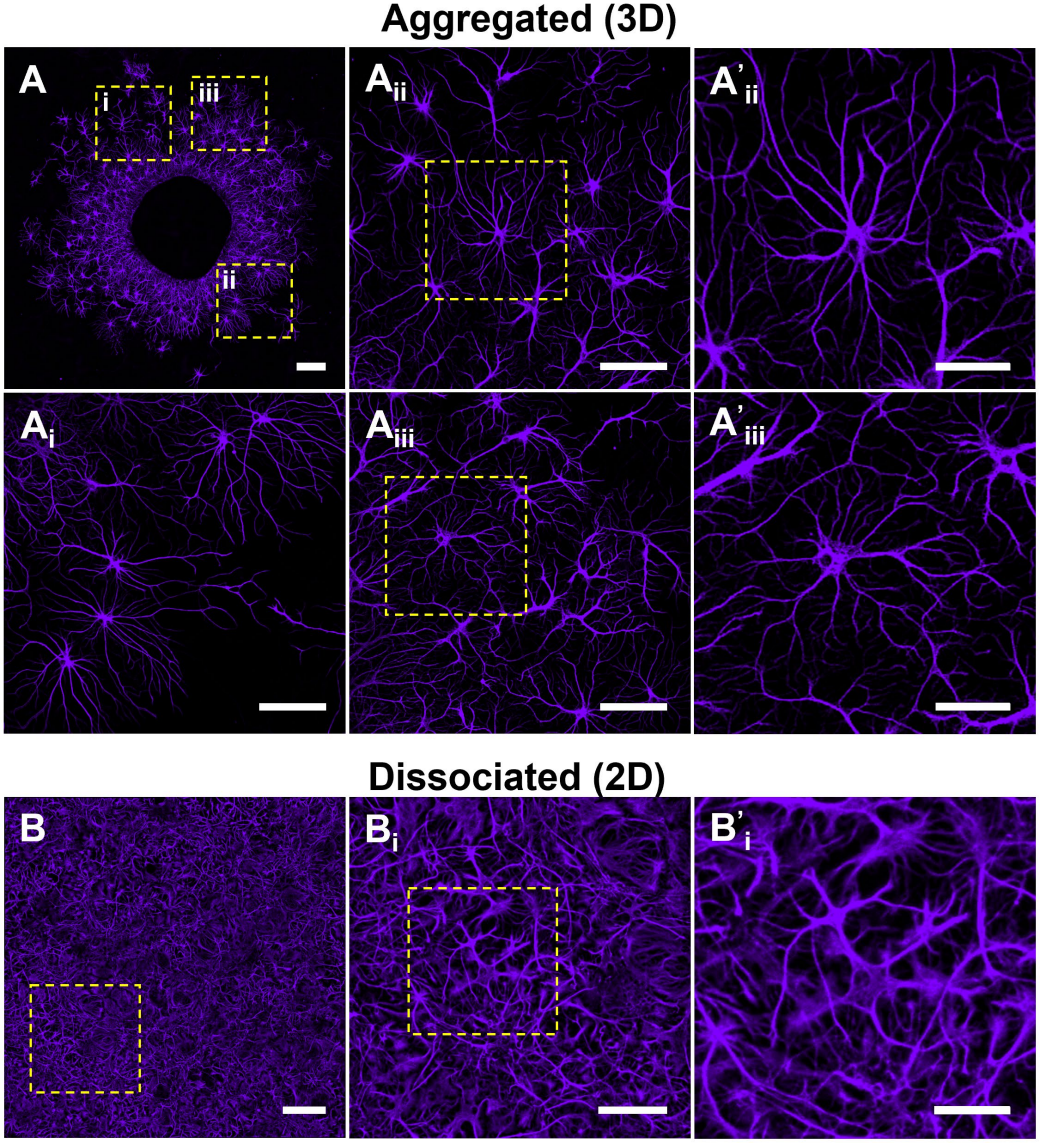
Astrocytes in aggregated cultures self-organize into non-overlapping domains. We qualitatively assessed astrocyte distribution and putative domain formation within aggregated versus dissociated cultures. Astrocytes in aggregated cultures were observed to organize into individual domains spatially-distanced from neighboring astrocytes. **(A)** Astrocytes demonstrated more apparent boundary formation as a function of distance from the aggregate. **(B)** In contrast, astrocytes in dissociated 2D cultures did not display a segregated spatial organization nor the formation of distinct borders between cells. **(A,B)** Scale bar = 200 μm; **(A**_i–iii_**)**, **(B**_i_**)** scale bar = 100 μm; **(A’**_i,ii_**,B**^’^_i_**)** scale bar = 50 μm.

Astrocytes survived and exhibited robust growth in both dissociated and aggregated cultures despite the lack of media supplementation specific for astrocytes (G5 or serum-enriched media). We initially qualitatively assessed astrocyte domain formation among aggregated and dissociated hippocampal cultures. Astrocytes in aggregated cultures migrated from the aggregate body and self-organized into individual domains with peripheral contact yet spatially distanced from neighboring astrocytes. By extending processes within exclusive territories, the aggregate-derived astrocytes formed a tiling pattern with minimal overlap with the domains of adjacent cells (Figure 5A). The overall coverage of this astrocyte tiling within the culture increased over time as astrocytes systematically migrated away from the aggregate. Astrocytes also demonstrated more distinct boundary formation as a function of distance away from the aggregate. In contrast, astrocytes in dissociated cultures displayed considerable process overlap and interdigitation between neighboring astrocytes (Figure 5B). To confirm that there were no density differences between culture conditions which might be mediating the domain-like and highly arborized morphologies of astrocytes, we conducted an evaluation of astrocyte density in both 2D cultures and within distinct zones of the 3D aggregate cultures (Supplementary Figure 4). We determined that there was no significant difference in astrocyte density between 2D and 3D cultures. The only significant difference in astrocyte density was between Zone 1 and 3 in 3D cultures (Supplementary Figure 4).

**Figure 6:**
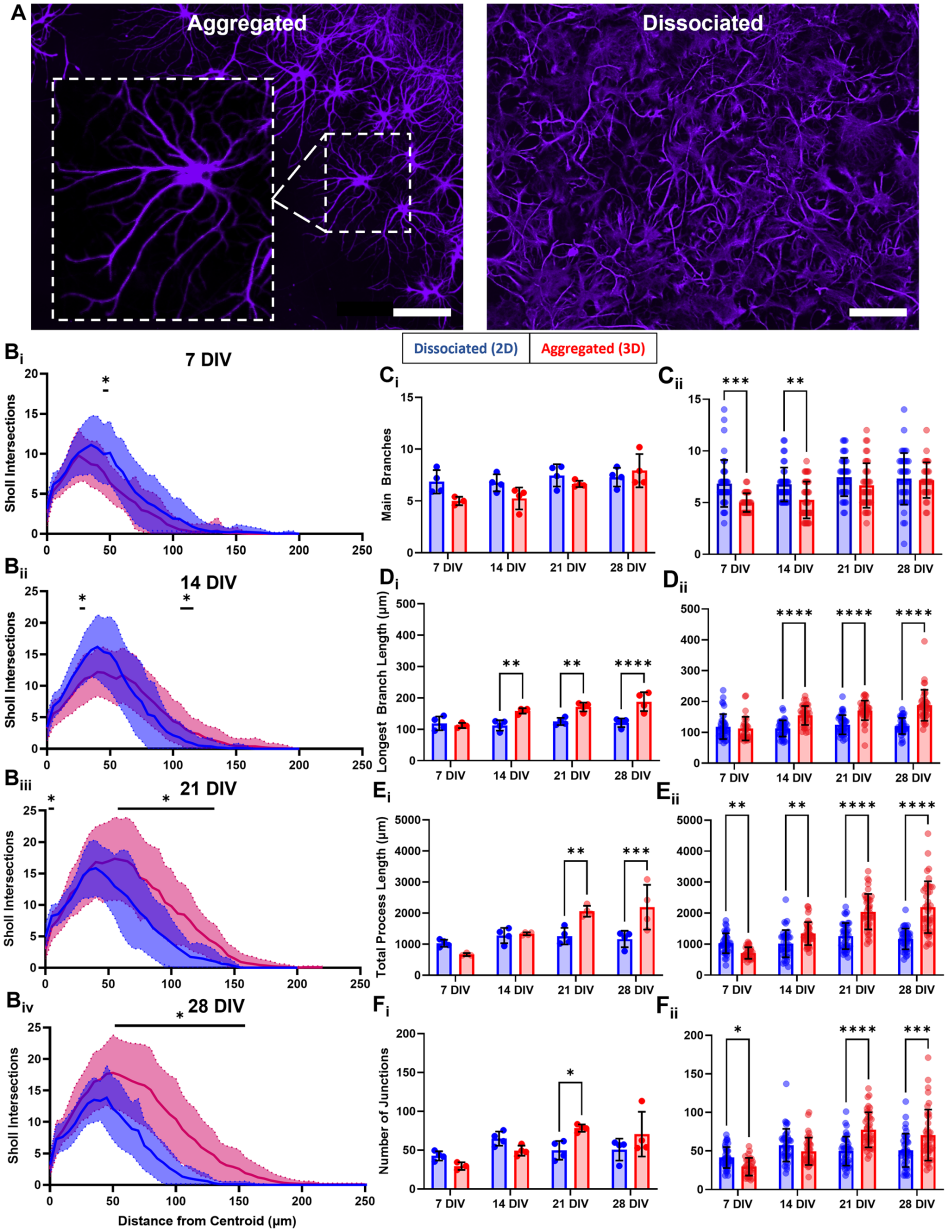
Astrocytes develop more arborized, stellate morphologies in aggregate cultures. **(A)** Following our observations that astrocytes in aggregated and dissociated cultures developed qualitatively distinct morphologies. **(B)** We adapted the Sholl analysis of neuronal arborization to the quantification of astrocytic arborization in each culture, revealing significant differences in astrocyte arborization in 2D versus 3D at specific distance ranges (7 DIV, 55–60 μm; 14 DIV, 45 and 120–130 μm; 21 DIV, 5 and 90–155 μm; 28 DIV, 60–170 μm). **(C**_i_**)** Average number of main, or primary, branches per astrocyte in dissociated and aggregated cultures. **(C**_ii_**)** Number of main branches for each individual astrocyte (7 DIV, *p* = 0.0004; 14 DIV, *p* = 0.0019). **(D**_i_**)** Average length of longest branch (14 DIV, *p* = 0.03; 21 DIV, *p* = 0.03; 28 DIV, *p* < 0.0001). **(D**_ii_**)** Length of the longest main branch for each individual astrocyte (14, 21, and 28 DIV, *p* < 0.0001). **(E**_i_**)** Mean total process length per astrocyte (21 DIV, *p* = 0.0047; 28 DIV, *p* = 0.0005). **(E**_ii_**)** Total process length for each individual astrocyte (7 DIV, *p* = 0.007; 14 DIV, *p* = 0.0052; 21 and 28 DIV, *p* < 0.0001). **(F**_i_**)** Mean number of junctions, or branch points, per astrocyte (21 DIV, *p* = 0.0008). **(F**_ii_**)** Number of junctions for each individual astrocyte (7 DIV, *p* = 0.0423; 21 DIV, *p* < 0.0001; 28 DIV, *p* = 0.0001). Scale bar = 100 μm. The significance levels were denoted as follows: * for *p* < 0.05, ** for *p* < 0.01, *** for *p* < 0.001, and **** for *p* < 0.0001.

Our observations suggested that astrocytes in aggregated cultures developed more arborized and stellate morphologies (Figure 6A). To quantify potential differences in astrocyte morphology, we constructed Sholl curves using the Neurite Tracer Tool, which plot the number of intersections processes make through a concentric ring at increasing distances from the astrocyte centroid (refer to Supplementary Figure 1). The resulting curves were used to quantify arborization and branching of astrocytes in aggregated and dissociated cultures at 7, 14, 21, and 28 DIV. At each time point, we observed a quantitative difference in the morphology of astrocytes between the two culture conditions. Specifically, we found that astrocytes in aggregated cultures had more crossings at greater distances compared to those in dissociated cultures (Figure 6B). Additionally, we observed a trend where the range of distances with significantly greater crossings for astrocytes in aggregated cultures increased over time, with the following distance ranges showing significant differences: 55–60 μm at 7 DIV, 45 μm and 120–130 μm at 14 DIV, 5 μm and 90–155 μm at 21 DIV, and 60–170 μm at 28 DIV (Figure 6B). We also noted that the intersection maximum at mature timepoints of 21 and 28 DIV was greater for astrocytes in aggregated cultures. These results suggest that astrocyte morphology is affected by the culture condition, and that astrocytes from aggregated cultures develop more arborized morphologies.

We also extracted and compared morphometric parameters between culture conditions, e.g., number of main branches, length of longest main branch, total process length, and number of junctions per astrocyte. We used two distinct approaches for statistical analysis of these morphological outcomes. First, we used a *culture-based* analysis, in which we averaged the values across the astrocytes in each culture. Next, we used a *cell-based* analysis, where individual astrocyte values where used and we did not average the values across astrocytes for a given culture, to better represent the biological variability within cultures. We quantified the number of astrocyte main branches, i.e., branches originating at the astrocyte soma. Culture-based analysis revealed no significant differences between groups across all timepoints (Figure 6C_i_). Cell-based analysis showed that astrocytes in dissociated cultures had a significantly greater number of main branches per astrocyte at 7 DIV and 14 DIV, however, this difference disappeared at 21 and 28 DIV (Figure 6C_ii_). By 21 DIV, across culture conditions, the average number of main branches per astrocyte was approximately 6–7. Both analysis approaches revealed that only astrocytes in aggregated cultures significantly increased in the number of main branches between 7 DIV and 28 DIV. Next, we assessed differences in the length of the longest main branch. Culture-based analysis showed that astrocytes in aggregated cultures had significantly longer “longest main branch” at 14, 21, and 28 DIV, with the greatest difference at 28 DIV (Figure 6D_i_). Cell-based analysis similarly showed that astrocytes in aggregated cultures had a significantly longer “longest main branch” at 14, 21 and 28 DIV (Figure 6D_ii_). For both analysis approaches, we found that for astrocytes in dissociated cultures this measurement remained similar across DIV, while in aggregated cultures it increased significantly between 7 DIV and 14, 21, and 28 DIV. Next, we assessed the total process length per astrocyte, which was the cumulative sum of all the main, secondary, and tertiary branches for an astrocyte. Culture-based analysis showed total process length for astrocytes in aggregated cultures was significantly greater at 21 and 28 DIV (Figure 6E_i_). Cell-based analysis showed total process length per astrocyte in aggregated cultures was significantly greater at all time points but showed greater differences at 21 and 28 DIV (Figure 6E_ii_). Both culture-based and cell-based analysis showed that the total process length of astrocytes in dissociated cultures remained similar across DIV, while in aggregated cultures it increased significantly between early (7 and 14 DIV) and late timepoints (21 and 28 DIV). In aggregated cultures, the average total process length increased nearly 3-fold from 713.4 ± 185.2 μm at 7 DIV to 2115.9 ± 834.5 μm at 28 DIV. Finally, we measured the number of junctions, i.e., points at which new branches form and diverge, as a correlate of arborization. Culture-based analysis showed that the number of branch junctions per astrocyte in aggregated cultures was significantly greater at 21 DIV (Figure 6F_i_). Cell-based analysis showed the number of branch junctions per astrocyte in aggregated cultures was significantly greater at 21 and 28 DIV (Figure 6F_ii_). Both culture-based and cell-based analysis showed the number of junctions per astrocyte in dissociated cultures was relatively consistent across DIV, while in aggregated cultures there was a significant ~2.5-fold increase between 7 and 21–28 DIV.

### 3.4. Aggregate networks develop unique electrophysiological profiles

**Figure 7:**
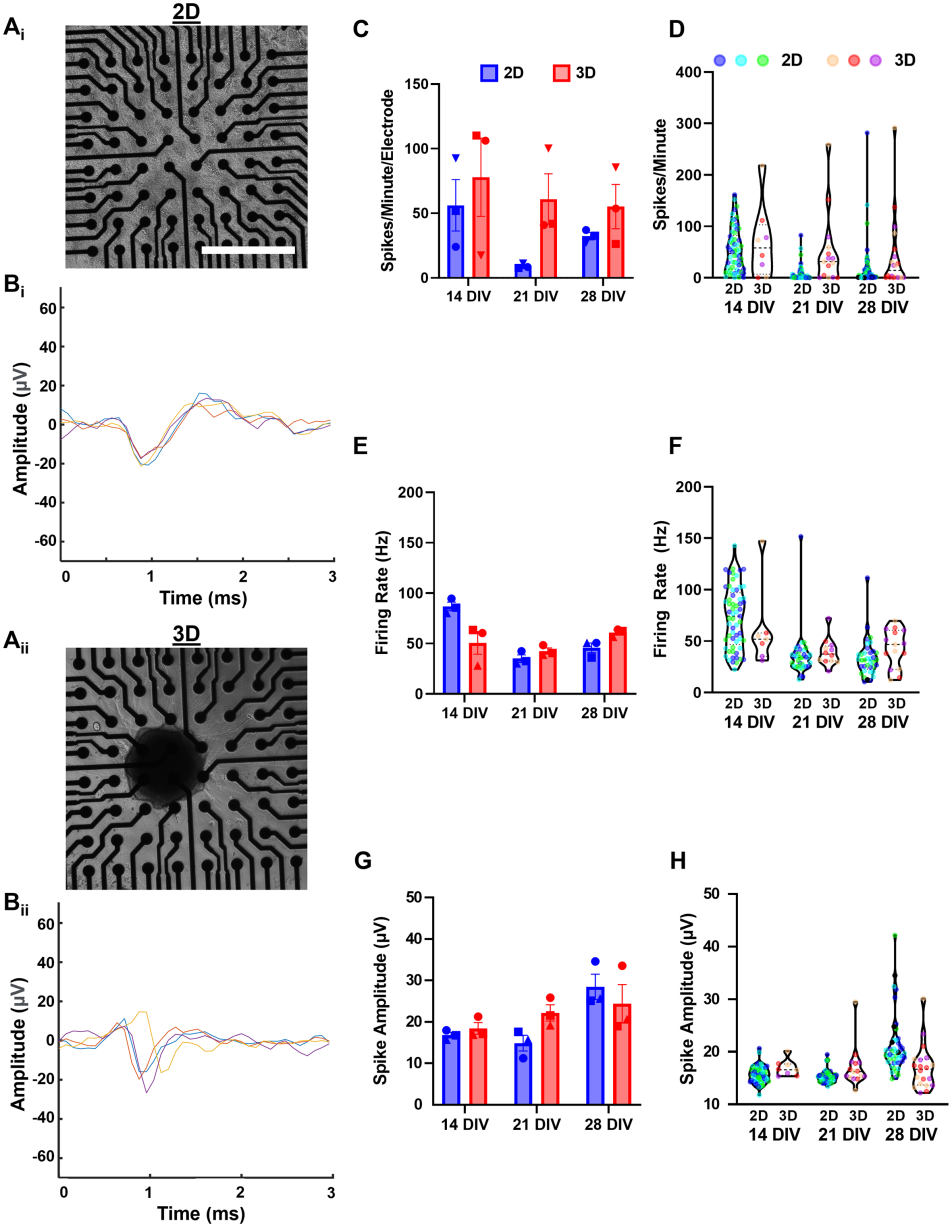
Assessment of spontaneous spiking activity shows hippocampal aggregates develop active networks. **(A**_i_**)** Aggregated and **(A**_ii_**)** dissociated cultures were imaged at 7 DIV before the start of experiments. **(B**_i,ii_**)** Representative plots of four consecutive spike waveforms from 2D and 3D networks demonstrating that our spike detection algorithm is effectively isolating individual spikes. **(C)** Spikes per minute per electrode across cultures and **(D)** spikes per minute values for individual electrodes from each culture. **(E)** Mean firing rate (Hz) across cultures and **(F)** mean firing rate (Hz) for individual electrodes from each culture. **(G)** Finally, mean spike amplitude across cultures and **(H)** mean spike amplitude for individual electrodes from each culture. Scale bar = 500 μm.

We cultured dissociated (2D) and aggregated (3D) networks on 64-electrode multi-electrode arrays MEA (Figure 7A_i-ii_) and recorded spontaneous activity at 7, 14, 21, and 28 DIV. Single aggregate cultures showed few active electrodes at 7 DIV, suggesting that neuronal aggregates required more time to spread and form contacts along the planar MEA surface or that 3D networks develop measurable activity more gradually. We conducted a preliminary visual examination of the spike waveforms of consecutive spikes from representative electrodes in both 2D and 3D networks to confirm that our spike detection algorithm was functioning appropriately. The representative waveforms from the 2D network are presented in Figure 7B_i_ and those from the 3D network in Figure 7B_ii_.

For all quantified outcome measures, we calculated the mean across all active electrodes *per culture* and used those values to assess statistical significance. To provide an assessment of variability across all electrodes, we also report the values for each *individual electrode* across all cultures within a group, however, we did not perform statistical analysis with these values. Here, we found no significant between-group or within-group differences in spikes per minute per electrode across timepoints (Figure 7C). Although not significant, 3D networks were likely to have greater spikes per minute per electrode at any measured timepoint (Figures 7C,D). We also quantified the mean firing rate (Hz), but did not find significant between-group differences at any timepoints (Figures 7E,F). However, we found that 2D cultures had a significant decrease in the firing rate from 14 DIV to 21 DIV (mean difference of 51.38 Hz) which persisted until 28 DIV (mean difference 41.03 Hz). In contrast, 3D networks had a significant increase in firing rate between 21 and 28 DIV (mean difference 18.37 Hz). Finally, we did not find any significant between-group or within-group differences in mean spike amplitude Left.

**Figure 8:**
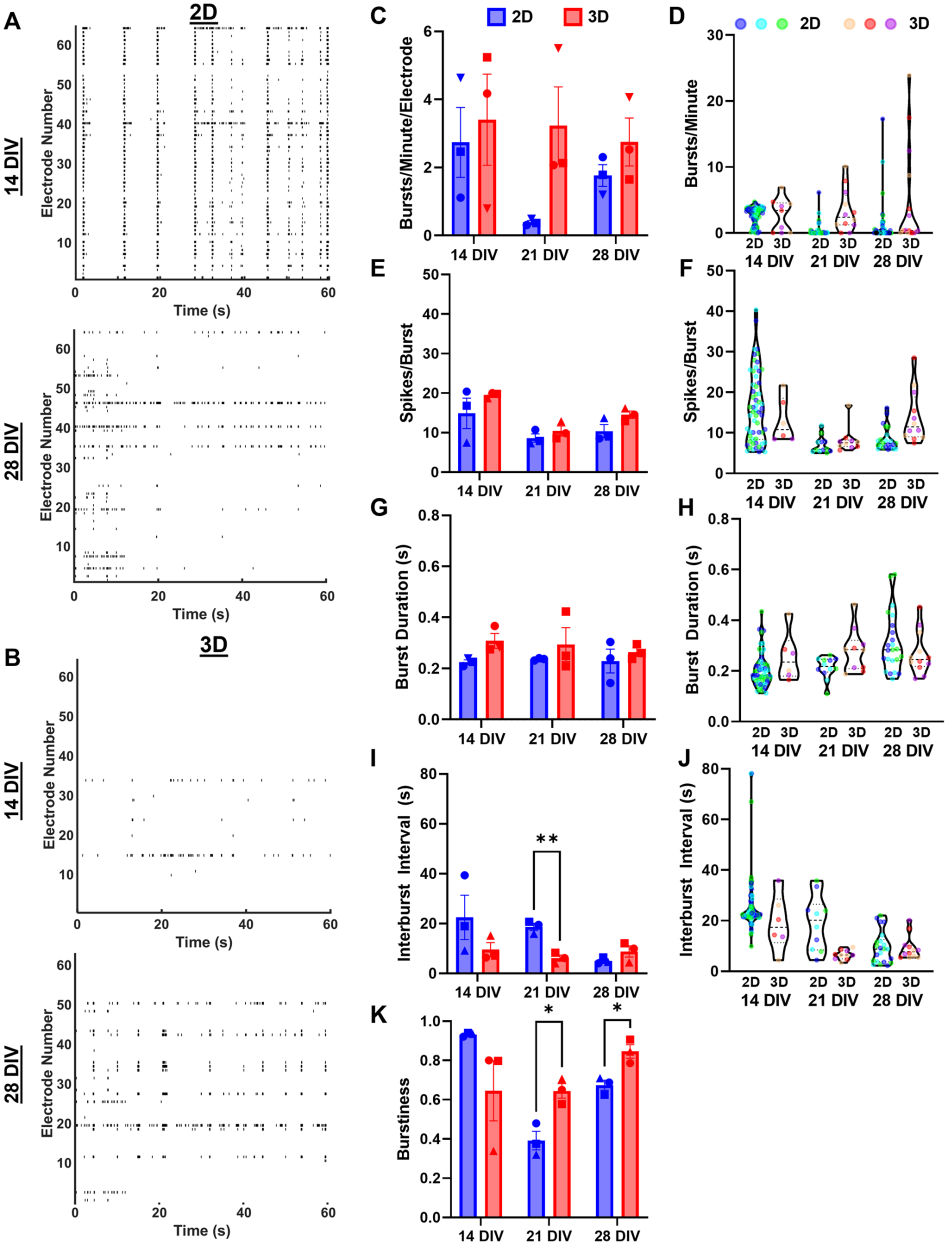
Assessment of spontaneous bursting activity reveals distinct bursting characteristics between 2D and 3D networks. **(A,B)** Raster plots of representative samples of network activity at 14 and 28 DIV for dissociated and aggregated cultures. **(C)** Bursts per minute per electrode across cultures and **(D)** bursts per minute values for individual electrodes from each culture. **(E)** Spikes per burst across cultures and **(F)** spikes per burst for individual electrodes from each culture. **(G)** Burst duration across cultures and **(H)** burst duration for individual electrodes from each culture. **(I)** Interburst interval across cultures (21 DIV, *p* = 0.0088) and **(J)** interburst interval for individual electrodes from each culture. **(K)** Finally, the burstiness per culture, i.e., proportion of total spiking activity occurring within a burst (21 DIV, *p* = 0.0430; 28 DIV, *p* = 0.0430). The significance levels were denoted as follows: * for *p* < 0.05, ** for *p* < 0.01.

Next, we characterized the bursting characteristics of 2D and 3D networks at 14, 21, and 28 DIV. We found that 2D networks developed highly synchronized and regular bursting activity by 14 DIV, but often developed less synchronized and regular bursting by 28 DIV (Figure 8A). In contrast, 3D networks developed more synchronized bursting as they matured from 14 to 28 DIV (Figure 8B). We found that 3D networks trended toward having greater bursts per minute per electrode (i.e., bursts per minute normalized to the number of active electrodes) than 2D networks at all timepoints, but this difference was not statistically significant (Figure 8C). While there was noticeable drop in the bursts per minute per electrode for 2D networks from 14 to 21 and 28 DIV, we did not find any significant within-group changes across (Figure 8D). We assessed the structure of individual bursts by measuring spikes per burst and burst duration. We found no significant differences between 2D and 3D networks at any timepoint (Figure 8E). While we found that in 2D networks spikes per burst remained similar across time points, it increased significantly in 3D networks between 21 and 28 DIV. We found no significant changes in burst duration in 2D or 3D networks across timepoints. Next, we examined the temporal structure of burst ensembles by measuring the interburst interval (IBI). We found that the mean IBI was significantly higher in 2D networks at 21 DIV (Figure 8I). The mean IBI was stable for 3D networks, but we found a significant decrease at 21 DIV for 2D networks. Finally, we measured burstiness, i.e., the ratio of number of spikes within-bursts to the total number of spikes (if all spiking occurred in bursts, burstiness would be equal to 1). We found 3D networks had significantly higher burstiness, relative to 2D networks, at 21 and 28 DIV (Figure 8K). Although not significant, 2D networks had the highest burstiness at 14 DIV (mean = 0.934) relative to 2D or 3D networks at any timepoint. Moreover, burstiness in 2D networks changed significantly across each timepoint, while it remained relatively stable in 3D networks.

One notable constraint of the current study is the absence of spike sorting algorithms in our approach. As a result, our findings predominantly reflect population activity and cannot be directly ascribed to alterations in individual neuronal properties. For example, differences in firing rates could be a function of increased neuronal excitability at the individual level, or alternatively, a rise in the overall number of neurons exhibiting spiking activity.

### 3.5. Dual-aggregate networks develop coordinated activity and bursting motifs

**Figure 9:**
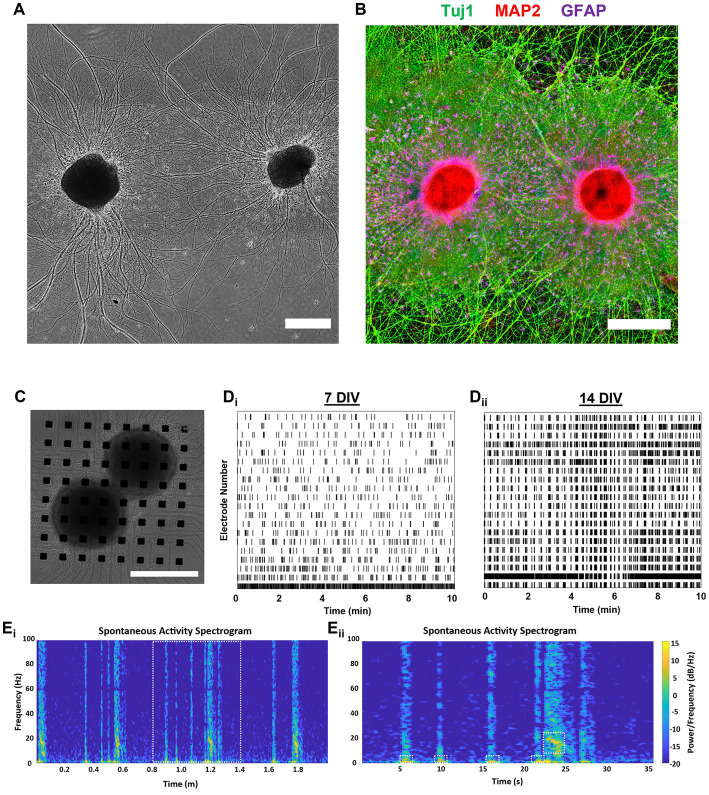
Assessment of dual-aggregate network activity shows rapid maturation and relevant electrophysiological properties. **(A,B)** Dual-aggregate cultures support long-distance axonal fasciculation, neuronal polarization, and quasi-domain formation by astrocytes, and were not morphologically differentiable from single aggregate cultures. **(A)** Representative phase image of a dual-aggregate system at 14 DIV. **(B)** We used immunocytochemistry to visualize the axonal processes, somatodendritic compartments, and astrocytes domains of a representative dual-aggregate culture at 21 DIV. **(C)** Representative culture featuring two aggregates on (generally) separate corners of a 64-electrode MEA for 14 DIV. Raster plots of a continuous 10-min recording from a representative dual-aggregate network demonstrating that dual-aggregate systems develop more synchronized bursting activity as they mature from **(D**_i_**)** 7 DIV to **(D**_ii_**)** 14 DIV. **(E**_i_**)** To visualize the oscillatory dynamics of the dual-aggregate system we generated a spectrogram (0–100 Hz) of a 2 min recording. **(E**_ii_**)** Zoom-in of a representative super-burst composed of preceding short duration bursts with low frequency bursting (2–10 Hz) activity followed by a longer duration burst dominated by higher frequency bursting (10–20 Hz); various bursting regimes denoted by dashed line boxes. **(A–C)** Scale bar = 500  m.

We used phase imaging to qualitatively assess the morphological maturation of double-aggregate cultures over time, and found these cultures also developed large long-distance axon fascicles by 14 DIV (Figure 9A). We also used immunocytochemistry to confirm that dual-aggregate cultures exhibited similar morphological phenomena to single-aggregate cultures, i.e., astrocyte arborization and domain-like self-organization, neuronal polarization, and robust fasciculation were recapitulated across within dual-aggregate cultures (Figure 9B). To examine the functionality of dual-aggregate networks, we cultured two hippocampal aggregates on diagonally separate corners of 64-electrode MEAs for up to 14 DIV (Figure 9C). Raster plots of a continuous 10-min recording at 7 and 14 DIV shows that networks increase overall activity, bursting, and synchronization across electrodes (Figure 9D). Notably, while single aggregate cultures lacked measurable spontaneous activity at 7 DIV, we found that dual-aggregate cultures showed substantial spiking activity by 7 DIV. We also constructed representative spectrograms (0–100 Hz) of spontaneous activity to visualize the oscillatory dynamics of the dual-aggregate system (Figure 9E_i_). We observed relatively stable patterns of bursting, where a series of short duration bursts with greater power in 2–10 Hz range would precede a long duration burst with greater power in the 10–20 Hz range (Figure 9E_i_). We highlight the temporal evolution of one of these so-called “super bursts,” appearing as a cluster of numerous successive bursts with greatest power in the 10–20 Hz range, which were consistently preceded by more spread out, short, rapid bursts dominated by lower frequency activity in the 2–10 Hz range (as denoted by dashed line boxes in Figure 9E_ii_).

## 4. Discussion

**Figure 10:**
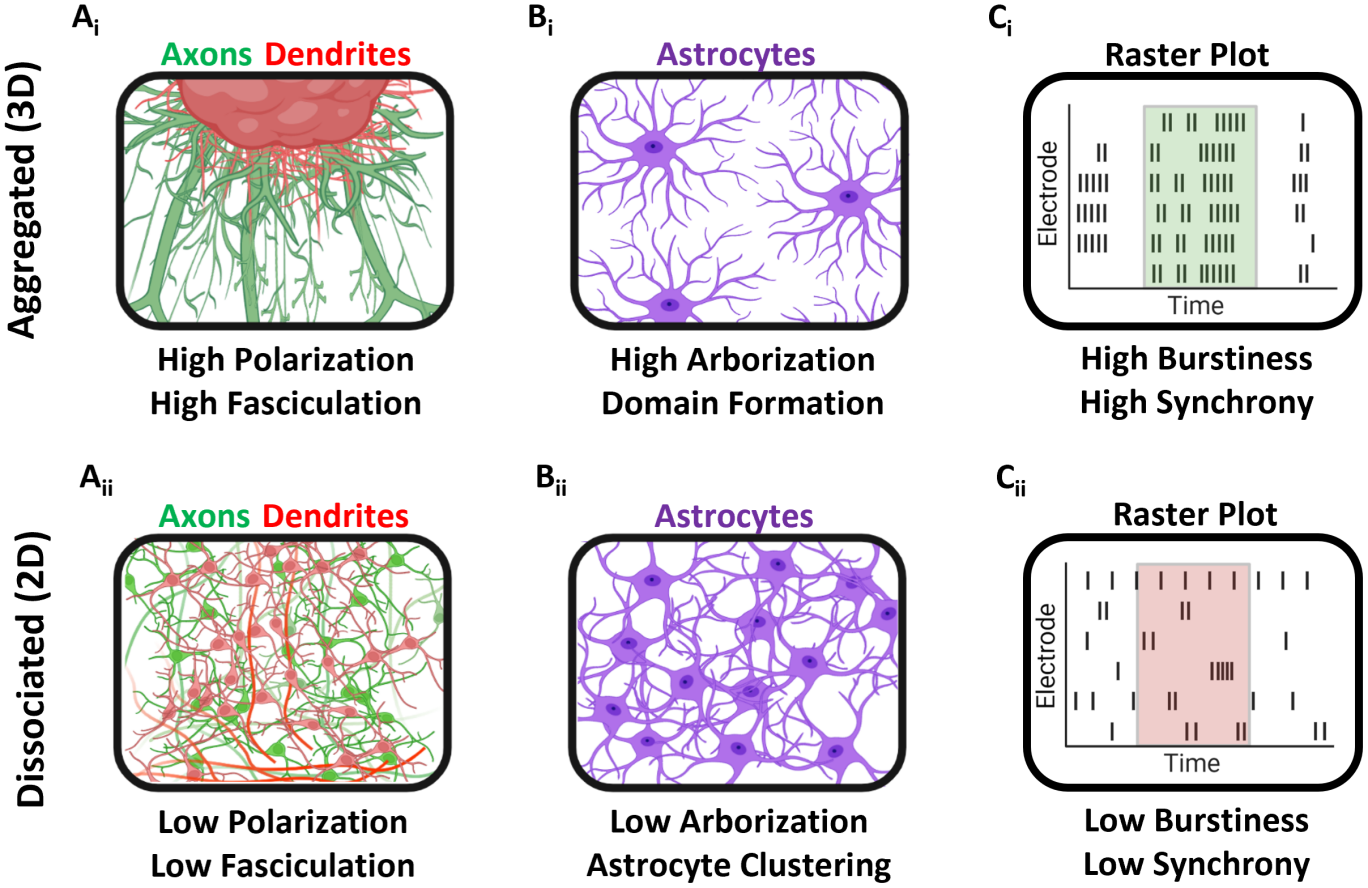
Graphical summary of findings. **(A**_i_**)** Aggregated cultures developed large axonal fascicles and demonstrated neuronal polarization (distinct dendrite and axonal spatial localization). **(A**_ii_**)** In dissociated cultures, axonal fasciculation and neuronal polarization were greatly attenuated. **(B**_i_**)** Astrocytes in aggregate cultures self-organized into domain-like topologies and demonstrated robust arborization relative to dissociated cultures. **(B**_ii_**)** Dissociated culture astrocytes showed high spatial clustering and low arborization. **(C**_i,ii_**)** Finally, aggregated cultures grown on MEAs had greater burstiness, i.e., proportion of total spiking activity occurring within a burst, at 28 DIV, as compared to dissociated cultures.

In the present study, we compared the properties of 2D dissociated and 3D aggregated cultures of hippocampal tissue to investigate the effect of three dimensionality on neuronal-glial development morphologically and functionally. Neuronal polarization, a key feature of endogenous brain networks, was enhanced in aggregate cultures, where MAP2+ somatodendritic compartments were found proximal to the aggregate and had minimal overlap with Tuj1+ axonal compartments (Kosik and Finch, 1987; Kanaan and Grabinski, 2021; van Niekerk et al., 2022). The axonal projections in aggregate cultures also demonstrated greater axonal fasciculation relative to dissociated cultures, more closely resembling the long distance white matter pathways of the brain (Barry et al., 2010; Wakade et al., 2013). Astrocytes in aggregate cultures also spontaneously self-organized into non-overlapping astrocyte domains and demonstrated high degrees of arborization, similar to the morphologies of astrocytes *in vivo* (Freeman, 2010; Robertson, 2018). We also demonstrated that 3D networks developed highly synchronized bursting activity by 28 DIV, in contrast to 2D networks which showed decreases in synchronized bursting activity over time. We also found that 3D networks had higher burstiness relative to 2D networks. Finally, analysis of dual-aggregate network electrophysiology revealed stable patterning of bursts at DIV 14. These 3D, high-density, multi-cellular aggregates closely recapitulate elements of the neuronal microenvironment for appropriate cellular and whole-tissue maturation, which we demonstrated could drive more phenotypically appropriate and biofidelic morphological and electrophysiological maturation in the aggregate network. The main findings and distinguishing features between dissociated and aggregated cultures are summarized in Figure 10.

Our forced aggregation method created neuronal aggregates that have densities mimicking native brain tissue (>100,000 cells/mm^3^), which may mitigate the morphological and functional impairments that can arise from insufficient densities (<10,000 cells/mm^2^) that are common in most planar culture models. In fact, culture density has been shown to be inversely correlated with the rate of network bursts and positively correlated with local burst rate (Cohen et al., 2008). Culture density also inversely influences the synapse-to-neuron ratio (Cullen et al., 2010). However, even ultra-low density (300–1,500 cells/mm^3^) neuronal cultures in 3D hydrogels generate stable and diverse bursting patterns consisting of short high frequency bursts, while 2D cultures demonstrate more prolonged and disorganized periods of fast spiking (Frega et al., 2014). Native brain networks rely on 3D connectivity and organization to produce dynamic repertoires of oscillatory activity (Ulloa Severino et al., 2016). In fact, hippocampal slice cultures below 350 μm are nearly incapable of generating sharp wave ripples; this suggests a critical threshold for network size in 3D and not just 2D. Our neuronal aggregates (4.3–6.5 × 10^7^ μm^3^) enable appropriate neuronal development and self-organized connectivity in 3D, thus more closely reconstituting the native microenvironment. Aggregated cultures were shown to produce robust fascicles, emanating from the aggregate body, which would span long distances (Figure 4 and Supplementary Figure 2). Such robust fascicles are not observed in dissociated cultures. Establishment of axonal projections from a target is a multistep process driven by various molecular cues (Šmít et al., 2017); we suspect the aggregate microenvironment creates a gradient in molecular cues (within and outside the aggregate) that may drive the formation of axonal fascicles. Axonal fasciculation has been shown to be tightly regulated by the function of neuronal cell adhesion molecules (NCAMs) both *in vitro* and *in vivo*, and experimental ablation of fasciculation by impairing NCAM function has been demonstrated (Koyama et al., 2004; Barry et al., 2010; Wakade et al., 2013).

We also noted that qualitative morphological properties of neurite outgrowth from the aggregates were highly variable between cultures. For instance, some aggregated cultures demonstrated a more homogenous distribution of axonal projections around the perimeter of the aggregate, while others had more localized sites of explosive axonal growth (Supplementary Figure 2). Other unique patterns of growth were observed, such as a dense mesh of neurites condensing into a fascicle “skirt” around the aggregate, from which axons would then sprout (Figures 3D–F). Outgrowth between two aggregates was similarly unpredictable – we observed phenomena resembling both chemoattraction and chemorepulsion from one aggregate onto the other (Šmít et al., 2017). Biochemical gradient guidance is a common neurodevelopmental mechanism used to navigate growth cones during axonogenesis (Davis et al., 2017). This varied behavior is likely driven by the pan-hippocampal composition of our aggregates. *In vivo*, the subfields of the hippocampus, e.g., dentate gyrus, CA1, CA2, and CA3, are connected in a highly organized schema; the neuronal phenotypes of these subfields are present in E18 tissue that we utilized for our aggregates (Shetty and Hattiangady, 2013). So, neurite outgrowth from and between the aggregated neurons would be driven by a combination of intrinsic (i.e., neuron-specific growth characteristics) and extrinsic (e.g., chemoattractant and/or chemorepulsive cues) factors, which generally vary based on neuronal subtype and maturation. We also found that aggregated, but not dissociated, cultures exhibited neuronal polarization across all timepoints. Previous studies with dissociated cortical neurons showed that MAP2+ neurites would not fasciculate with Tuj1+ neurites, so attenuated neuronal polarization in dissociated cultures may mediate the reduced fasciculation we observed (Wakade et al., 2013). It is likely that the microenvironment-driven mechanisms that are promoting fasciculation in aggregates are likewise inducing neuronal polarization. However, we are unsure of the causal relationship between fasciculation and neuronal polarization in aggregated cultures. We quantified Mander’s Overlap Coefficients M1 and M2 as correlates for the extent of neuronal polarization (Figures 4B,C). Interestingly, in dissociated cultures there was large decrease in M1 and M2 at 14 and 21 DIV, which then reversed at 28 DIV. We believe that early increases in Tuj1+ axonal processes at 14 and 21 DIV decreased the proportional Tuj1/MAP2 signal colocalization, but that by 28 DIV dissociated cultures began to experience a loss of Tuj1+ axonal processes, which subsequently drove an increase in overall Tuj1/MAP2 colocalization. Aggregate test beds could be utilized as novel tools for exploring the processes regulating neuronal polarization in healthy and pathological neural systems, and how disruptions to neuronal polarization might affect other processes, such as axonal fasciculation. Axonal fasciculation in aggregate cultures could also be utilized to study abnormal axonal pathfinding and subsequent synaptogenesis in disorders such as autism where protein mutations can alter fascicle formation patterns and impact resultant axon pathfinding and network formation (Davis et al., 2017). This potential application could be pursued as well by using aggregates as nodes to construct networks and seeing how the disruption of axonal fasciculation affects emergent network properties and effective connectivity.

We found that our model produced astrocytes with a stellate morphology closely resembling *in vivo* quiescent astrocytes. It appears that the 3D cellular microenvironment of the aggregates was driving astrocytes to self-organize into quasi-domains and adopt highly ramified morphologies (Figure 5). Previous studies have demonstrated that elements of the microenvironment, such as dimensionality and matrix stiffness, are both capable of dramatically modifying astrocyte morphology (Balasubramanian et al., 2016; Hu et al., 2021). In our Sholl plots, we found that astrocytes in aggregated cultures, relative to dissociated cultures, had significantly greater intersections in specific regions across all time points (Figure 6B). Significant differences were mostly between 50 and 150 μm from the astrocyte centroid, and the region of significance increased from 5 μm (55 to 60 μm) at 7 DIV to 110 μm (60–170 μm) at 28 DIV. Astrocytes in aggregated cultures also trended toward more intersections at farther distances at all timepoints. In accordance with these results, cell-based, but not culture-based analysis, revealed significantly more main branches at early time points (7 and 14 DIV) for astrocytes in dissociated cultures. Culture-based analysis showed astrocytes in aggregated cultures had significantly greater longest branch length (at 14–28 DIV), total process length (at 21, 28 DIV), and number of junctions (at 21 DIV). Similarly, cell-based analysis showed astrocytes in aggregated cultures had even more significant differences in longest branch length (14–28 DIV), total process length (7–28 DIV), and number of junctions at mature time points (7, 21, 28 DIV). Notably, we found that astrocytes in dissociated cultures had nearly no significant within-group differences for any of the morphological metrics. The only exception being the significant increase in the number of junctions between 7 and 14 DIV revealed by cell-based analysis. This contrasts with astrocytes in aggregated cultures which showed significant within-group differences for each morphological metric. Overall, these results suggest that astrocytes in aggregated cultures develop more arborized main branches, i.e., more stellate morphologies. Given that the number of main branches is the same between conditions, we believe that the domain formation process is driving astrocytes to develop more ramified morphologies. In dissociated cultures, extensive interdigitation may be attenuating astrocyte stellation. We also confirmed that there were no differences in astrocyte density (astrocytes/mm^2^) between 2D and 3D cultures, thus this was ruled out a possible mechanism for astrocyte quasi-domains and stellate morphologies. We also observed preliminary boundary formation by astrocytes in hippocampal explants (Supplementary Figure 5D), which we believe further indicates that astrocyte quasi-domain formation in 3D cultures is initiated by the active migration of astrocytes from the body of the aggregate. Subsequent studies will attempt to further characterize the astrocyte domain formation phenomena. Moreover, we could investigate soluble factors potentially released differentially by aggregates relative to dissociated cultures in order to isolate the biochemical signaling underlying astrocyte ramification and domain formation. This information may increase the translational value and reliability of the results obtained from primary neural cultures.

We propagated hippocampal explants in culture and performed immunocytochemistry (ICC) at 7 DIV to assess their morphological characteristics (Supplementary Figure 5). Our analysis revealed that these explants display the same morphological features that we previously established as characteristic of hippocampal aggregates, such as fasciculation, polarization, and astrocyte domain formation. Importantly, the striking morphological similarity between hippocampal aggregates and explants affirms our position that our aggregation methodology effectively generates constructs that closely mimic the three-dimensional, high-density, multi-cellular micro-environment of native neuronal tissue. It is essential to highlight that our forced aggregation method offers unique advantages for the generation of 3D tissue constructs, relative to tissue explants, given its ability to allow precise control over cellular constituents. This includes, for example, the ability to modulate the ratio of various cell types (e.g., neurons to astrocytes) and/or implementing any genetic modifications or labels to specific cell types.

While morphological assessments of our aggregate model have supported the claim that aggregated cultures (i.e., 3D networks) are more biofidelic recapitulations of *in vivo* hippocampal networks relative to traditional dissociated cultures (i.e., 2D networks), we sought to validate whether these characteristics would likewise translate to functional (i.e., electrophysiological) outcomes. Most measures of activity for 2D networks showed significant decreases between 14 DIV and later timepoints (Figures 7C–G). Our lab’s previous work found that dissociated cultures exhibited an average 45% reduction in viability from 10 to 28 DIV, whereas aggregated cultures only exhibited a 15.2–18.6% drop over time, so it is possible that sudden decreases in activity are primarily mediated by the poor viability of dissociated cultures at later time points (Adewole et al., 2021). One of our objectives in assessing network activity was to establish that aggregates could attain mature timepoints (28 DIV) while exhibiting robust electrophysiological activity akin to endogenous hippocampal networks, characterized by synchronized activity and high burstiness. Overall, we observed few significant differences in the structure of bursts, e.g., spikes per burst, burst duration, and IBI (Figures 8E–J). We noted a non-significant trend toward more spiking and bursting per minute per electrode for 3D networks relative to 2D networks. More importantly, we were able to demonstrate that 3D networks, as compared to 2D networks, displayed increasingly synchronized activity and significantly greater burstiness at 21 and 28 DIV. These findings confirmed electrophysiological viability in the aggregate cultures out to 28 DIV as well as distinctiveness relative to dissociated culture networks. Although this evidence suggests that 3D cultures may be more similar to *in vivo* networks in terms of burstiness and synchrony, further comprehensive analyses of their network properties are required to determine their effectiveness to model more complex features of *in vivo* network activity.

While the present analysis was sufficient for determining differences between 2D and 3D neuronal networks, we believe that more robust analysis methodology could be utilized to produce more meaningful descriptions of 3D network activity. For instance, the representative waveforms in Figure 7B_i,ii_ are presented solely for the purpose of providing a visual representation of the data. The waveforms depicted are not meant to suggest any pattern or trend in terms of overlap, similarity, heterogeneity, or precision between 2D and 3D networks. Comprehensive quantitative analysis will be necessary in future work to make any definitive conclusions regarding potential differences in spike waveform characteristics between the two culture conditions. Moreover, the lack of absence of spike sorting algorithms in our analysis of network activity limits our understanding of changes in neuronal activity. For instance, without implementing spike sorting, it is not possible to determine whether changes in network firing rate are due to increases in single-neuron excitability or an increase in the overall number of spiking neurons. Subsequent endeavors will incorporate advanced spike sorting techniques, allowing us to delineate differences in population activity to variations in electrophysiological properties at the level of individual neurons. This will enable a more precise understanding of changes in network dynamics across the various model systems.

We sought to demonstrate that aggregates could be used as modular building blocks. To achieve this, we investigated the electrophysiological dynamics of a hippocampal dual-aggregate system. We found that the dual-aggregate networks became active by 7 DIV, whereas single aggregate systems did not develop substantial activity until 14 DIV. Thus, we suspect that multi-aggregate systems are potentiating maturation and development of the entire network. The temporal structure of a burst ensemble, or “super burst” (i.e., a series of bursts in close sequence), provides clues to the information coding space and internal network states that can be represented. So, we highlighted the temporal evolution of a single representative “super burst,” which followed a reliable pattern of short (~1 s) bursts with greater power in the 2–10 Hz range, followed by a single long (~4 s) burst with greater power in the 10–20 Hz range (Figure 9E_i,ii_). This relatively conserved pattern of activity might imply the presence of stable dynamic attractors in the oscillatory dynamics of the network, which is important for information processing at longer timescales. Similarly, *in vitro* cortical cultures have been shown to develop an extremely rich and varied repertoire of bursting patterns throughout development, with great burst pattern variability throughout development of a single culture and between cultures (Wagenaar et al., 2006). As such, in future studies we will attempt to examine the variability in the emergent patterns (temporal structure of super-bursts) of aggregate networks and attempt to identify reliably conserved features of the aggregate network dynamics by recording for longer (3+ hour) sessions. Moreover, in a multi-aggregate network, we would like to investigate the temporal dynamics between aggregates more closely to determine whether a single aggregate can act as a “leader” in the network to reliably trigger activity in another aggregate or synchronize activity across the entire network. This work will significantly enhance the value of this model for studying the myriad of distributed multi-node networks found in the brain.

A key limitation of this study is that the staining and/or imaging methods that we employed were not amenable to assessing the internal cellular distribution and/or morphology within the aggregates. For instance, the opacity of the thick aggregates prevented light microscopy examination beyond the outer surface, and SEM is inherently a surface visualization technique. In contrast, while confocal microscopy is a 3D imaging technique, additional immunocytochemical optimization would need to be performed to ensure consistent primary and secondary antibody penetration across the thickness of the aggregates. Therefore, future studies should explore the optimization of antibody penetration, tissue clearing, and/or tissue sectioning methodology in order to examine neuronal-glia cytoarchitecture, distribution, and growth within the aggregate structure over time.

Planar (2D) cultures generated from dissociated primary neuronal preparations are ubiquitous in neuroscience research, however due to their poor recapitulation of the native microenvironment, their emergent morphological and electrophysiological properties often differ in crucial ways from *in vivo* neuronal networks. The current work was primarily concerned with validating aggregate cultures as simple and high-throughput alternative to 2D cultures, due to their superior recapitulation of the neuronal microenvironment and, subsequently, its morphological and electrophysiological properties. There are many other model systems, such as human stem cell organoids and *ex vivo* slice cultures, which also have unique strengths and benefits over dissociated neuronal cultures. For instance, organoids offer incredible phenotypic and structural homology to the *in vivo* system being modeled; in particular, hippocampal organoids support the emergence of neuronal phenotypes and lamellar organization resembling hippocampal subfields *in vivo* (Sakaguchi et al., 2015; Ciarpella et al., 2021). Yet, even at mature timepoints of 3–6 months, they do not recapitulate adult electrophysiology and functionality (Tasnim and Liu, 2022). In contrast, primary neuronal cultures do develop adult phenotypes *in vitro* on the order of weeks in culture. However, this distinction allows organoids to be deployed as powerful models of fetal brain development. In addition, slice cultures are useful *ex vivo* models and are often used to address research questions where the network architecture needs to be exquisitely preserved, such as for the study of sharp wave ripple generation and propagation across hippocampal subfields. Simultaneously, the phenotypic and structural complexity of this model can also be unnecessary depending on the question being asked. Also, slice cultures are notably difficult to generate and maintain as there is significant trauma associated with tissue excision and often active degeneration caused by deafferentation and mass transport limitations – all of which would affect the fidelity of electrophysiological function over time. As such, there remains a complimentary role for using primary neuronal cultures in research, and our neuronal aggregate culture system should be seen as complimentary to these alternative model systems.

At present, it is unclear to what degree endogenous astrocytes were driving the electrophysiological maturation of aggregate networks. Given the importance of astrocytes for healthy synaptic function, it is likely that they are contributing to some degree. In future studies, we will attempt to compare the electrophysiological properties of aggregated cultures with and without endogenous astrocytes. Single aggregate cultures demonstrate great promise as models for pharmaceutical or electrophysiological investigations because they are effective approximations of the native high-density, multi-cellular, 3D microenvironment. However, the emergent properties (e.g., temporal patterning of activity) and functionality (e.g., learning) of neural networks are not *only* a consequence of the microenvironment, but also of the specific topology (modular organization) of the nodes composing the network. In fact, modular organization of dissociated cortical network nodes has been shown to directly modulate the dynamical richness of network activity (Yamamoto et al., 2018). Ultimately, aggregates made with various phenotypes, i.e., derived from specific brain regions or exhibiting specific neurotransmitters, could be utilized as modular building blocks to reconstruct more complex neuronal network topologies. We believe this study advances efforts in the development of more biofidelic multi-aggregate networks as tools for pharmacological and neurological investigations.

## 5. Conclusion

We demonstrated that a high-density, multi-cellular, 3D aggregate microenvironment can recapitulate critical emergent morphological and functional properties of *in vivo* networks. The aggregate model described here may be of interest to investigators because it can reliably and robustly induce neuronal polarization and fasciculation, as well as astrocyte arborization and quasi-domain self-organization, which are otherwise difficult to reproduce *in vitro*. Our assessment of spontaneous activity revealed that aggregate cultures, relative to dissociated networks, developed highly synchronized networks, and had higher burstiness at 28 DIV. We also demonstrated that dual-aggregate systems rapidly developed synchronized bursting activity and consistent bursting patterns by 14 DIV. This aggregate-based culture technique may be applied as “building blocks” to reconstruct modular neural network topologies *in vitro*. Various types of aggregates (e.g., cortical, hippocampal) could be arranged to resemble topologies of biological neural networks of interest, thus enabling highly controlled studies on the significance of topology in the emergent electrophysiology of a network.

Further, our findings suggest that in future studies neural aggregates may be used as segregated, modular building blocks for the development of complex, multi-nodal neural network topologies, toward the goal of mimicking the tri-synaptic pathway in the hippocampus critical for modeling the mechanisms underlying learning and memory formation. Indeed, we expect that hippocampal aggregates will be useful *in vitro* tools for neuroscientists to investigate the functional properties of neural networks in healthy and diseased states. Similar next-generation tissue engineering strategies will be crucial in the development of more efficacious and reliable pre-clinical screening tools. Future work will also be focused on the development of multi-aggregate cultures for the purposes of assessing the mechanism(s) of action underlying network development and responses to pharmacologic compounds.

## Data availability statement

The raw data supporting the conclusions of this article will be made available by the authors, without undue reservation.

## Author contributions

VA, SD, and DC were responsible for study design, interpretation of the results, and developed the final version of the manuscript. VA performed all experiments, acquired and analyzed all data, prepared all figures, and wrote the first draft of the manuscript. EP and DA assisted with cell culture and provided hippocampal tissue. OR assisted with cell culture, staining, imaging, and quantification. All authors contributed to interpretation of the results, manuscript revisions, approved of the final version, and agreed to be accountable for the content of the work.

## Funding

This work was supported by the National Science Foundation Graduate Research Fellowship Program (VA; Grant No. DGE-1845298), the National Institutes of Health (DC; NINDS R01-NS117757), and the Department of Veterans Affairs (DC; BLRD Merit Review I01-BX003748). Any opinions, findings, and conclusions or recommendations expressed in this material are those of the author(s) and do not necessarily reflect the views of the National Science Foundation, National Institutes of Health, or the Department of Veterans Affairs.

## Conflict of interest

DC was a scientific co-founder of Innervace, Inc., and Axonova Medical, LLC, which are University of Pennsylvania spin-out companies focused in neuroregenerative medicine.

The remaining authors declare that the research was conducted in the absence of any commercial or financial relationships that could be construed as a potential conflict of interest.

## Publisher’s note

All claims expressed in this article are solely those of the authors and do not necessarily represent those of their affiliated organizations, or those of the publisher, the editors and the reviewers. Any product that may be evaluated in this article, or claim that may be made by its manufacturer, is not guaranteed or endorsed by the publisher.
