# Supplementary material for: Emergent structural and functional properties of hippocampal multi-cellular aggregates
Source: Front Neurosci. 2023 Jun 15;17:1171115. doi: 10.3389/fnins.2023.1171115 (PMC10311220; doi:10.3389/fnins.2023.1171115)
Supplement: Supplementary file 1 [file Data_Sheet_1.PDF]

*Supplementary Material*

# Emergent Structural and Functional Properties of Hippocampal Multi-Cellular Aggregates

**Victor P. Acero[1,2,3], Suradip Das[1,2], Olivia Rivellini[1,2], Erin M. Purvis[1,2,4], Dayo O. Adewole[1,2,3], D. Kacy Cullen[1,2,3,4*]**

[1]Center for Brain Injury & Repair, Dept. of Neurosurgery, Perelman School of Medicine University of Pennsylvania, Philadelphia, PA, USA

[2]Center for Neurotrauma, Neurodegeneration & Restoration, Corporal Michael J. Crescenz Veterans Affairs Medical Center, Philadelphia, PA, USA

[3]Department of Bioengineering, School of Engineering & Applied Science, University of Pennsylvania, Philadelphia, PA., USA

[4]Department of Neuroscience, Perelman School of Medicine, University of Pennsylvania, Philadelphia, PA, USA

*** Correspondence:**
D. Kacy Cullen, Ph.D.
University of Pennsylvania
Department of Neurosurgery
105E Hayden Hall, 3320 Smith Walk
dkacy@pennmedicine.upenn.edu

## 1 Supplementary Figures

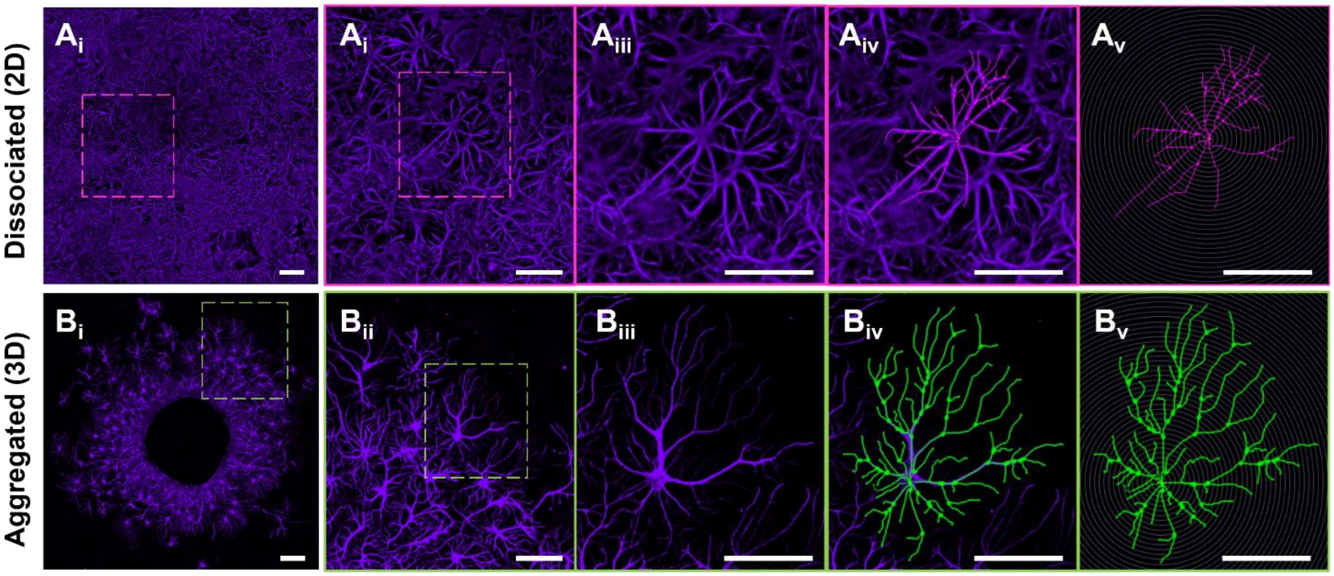


**Supplementary Figure 1.** Extracting morphometric data from astrocytes using the neurite tracer tool. We used the Simple Neurite Tracer (SNT) tool in ImageJ to semi-automatically hand-trace each astrocyte's arborization. We performed Sholl analysis originating at the center point of each soma to calculate how many times a circle of radius 5 units will intersect with the astrocyte's paths. $A_i$,$B_i$ scale bar = 200 μm; $A_{ii}$-$A_v$, $B_{ii}$-$B_v$, scale bar = 100 μm.

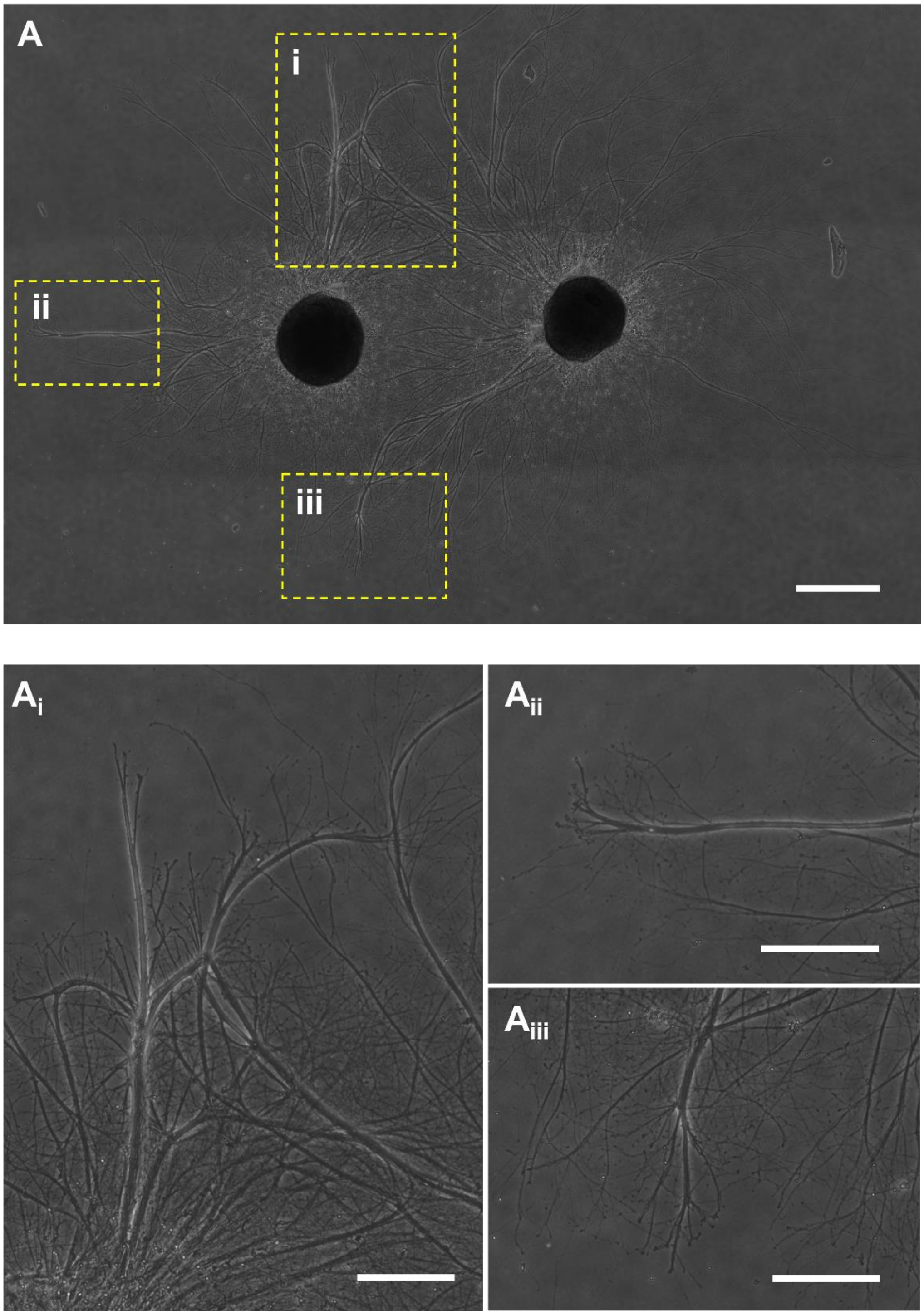


**Supplementary Figure 2.** Axonal outgrowth in dual-aggregate cultures. Phase contrast images of a representative dual-aggregate culture where each aggregate shows distinct axonal outgrowth characteristics **(A)**. Axonal fascicles are shown to growth towards, and away from, neighboring aggregates. We also show defasciculation of axonal projections and the "zippering" of multiple fascicles **($A_{i-iii}$)**. A, scale bar = 500 μm; B, scale bar = 250 μm.

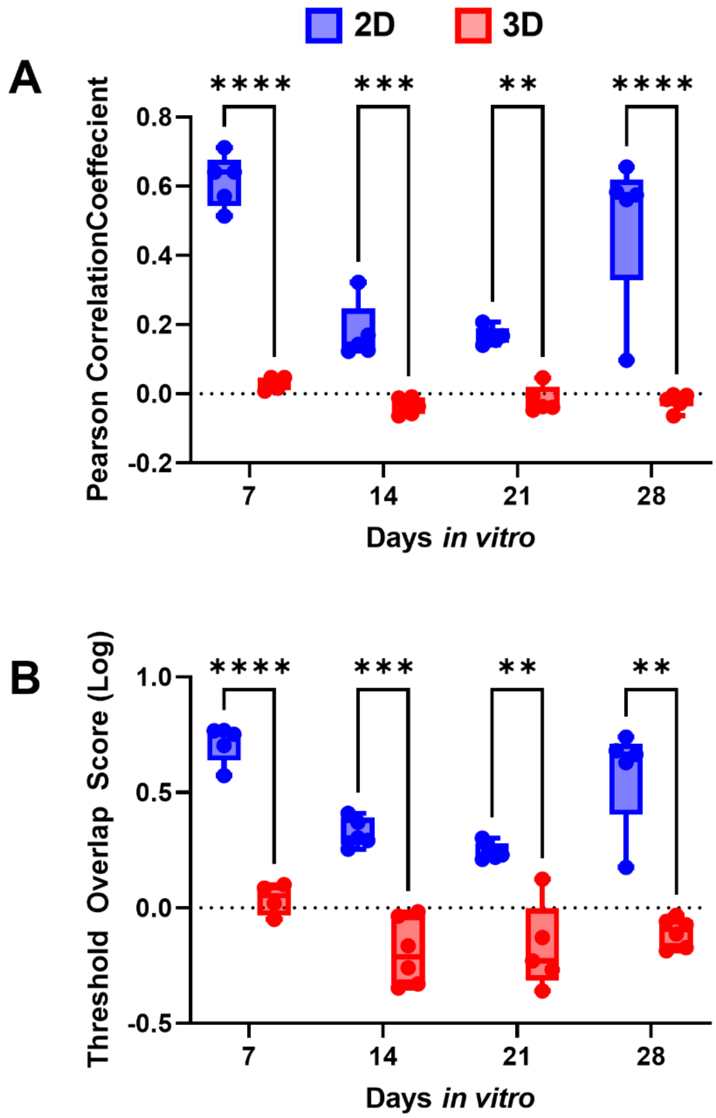


**Supplementary Figure 3.** Pearson Correlation Coefficients (PCC) and Threshold Overlap Score (TOS) for additional quantification of neuronal polarization. We assessed both the spatial overlap and co-labeling of neurites with Tuj1/MAP2 of aggregated and dissociated cultures. We quantified neuronal polarization between culture conditions across time utilizing the PCC (A) and TOS (B), which, unlike M1 and M2, account for signal intensity. PCC and TOS graphs show that dissociated cultures had significantly greater Tuj1 and MAP2 co-localization at all timepoints (For PCC: 7 DIV, $p < 0.0001$; 14 DIV, $p = 0.0009$; 21 DIV, $p = 0.0027$; 28 DIV, $p < 0.0001$. For TOS: 7 DIV, $p < 0.0001$; 14 DIV, $p = 0.0003$; 21 DIV, $p = 0.0065$; 28 DIV, $p = 0.0038$). The significance levels were denoted as follows: ** for $p < 0.01$, *** for $p < 0.001$, and **** for $p < 0.0001$.

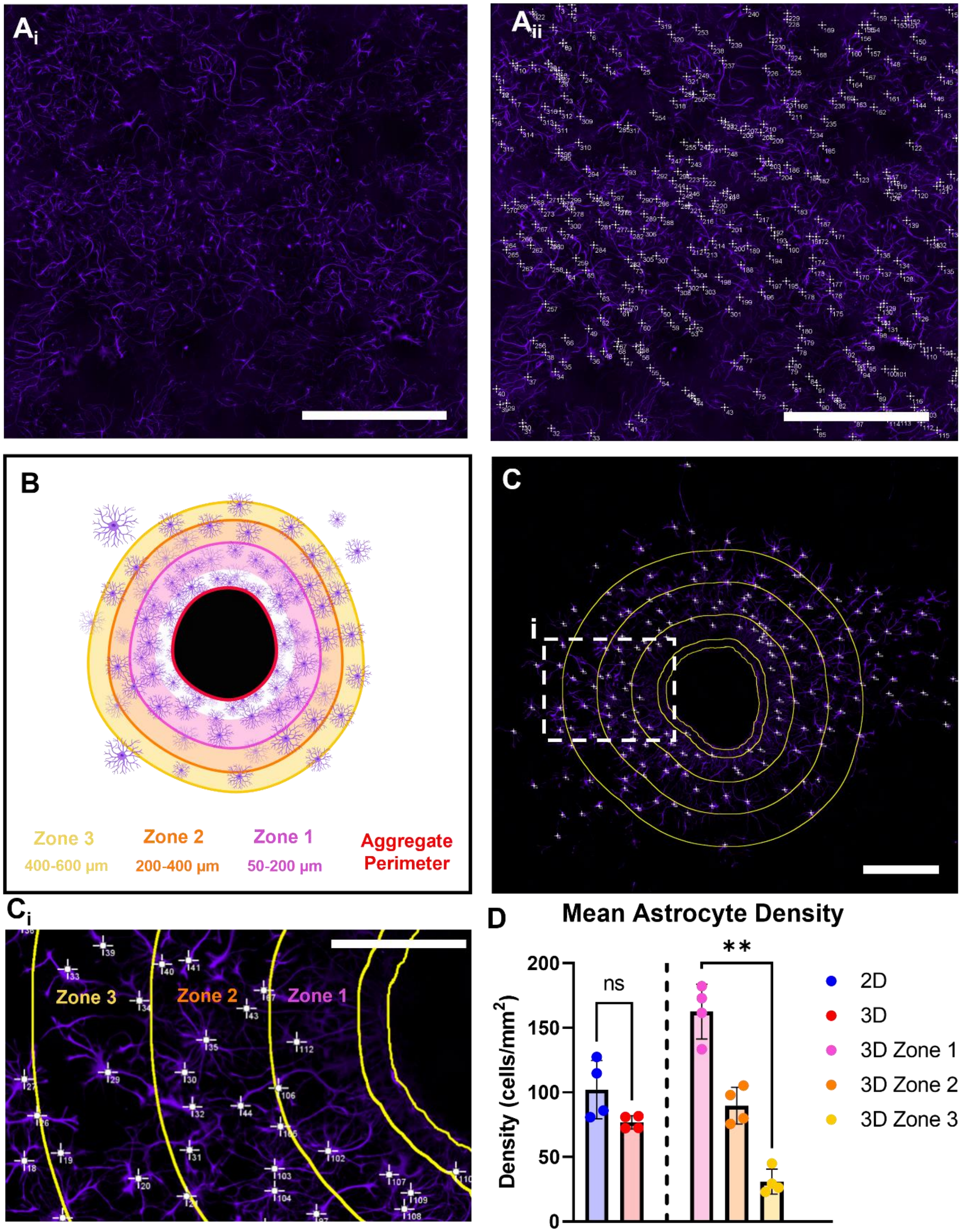


**Supplemental Figure 4:** Quantifying the mean astrocyte density in dissociated and aggregated cultures. ($A_{i\text{-}ii}$) We calculated the astrocyte density (cells/mm$^2$) in dissociated (2D) cultures at 7 DIV by manually counting in ImageJ. (B) Prior to quantifying the astrocyte density in aggregate (3D) cultures at 28 DIV, the aggregate culture area was divided into three concentric areas at

different distances from the aggregate perimeter: Zone 1 (50 – 200 µm), Zone 2 (200 – 400 µm), and Zone 3 (400 – 600 µm). (C) We then manually counted each astrocyte and calculated the astrocyte density across each Zone. ($C_i$) We also provide a magnified view of astrocytes from each of the zones in a representative 3D culture. (D) We plotted the mean astrocyte density in 2D and 3D cultures, and only found significant differences between Zone 1 and Zone 3 ($p = 0.0013$). (A, C scale bar = 500 µm; $C_i$ scale bar = 250 µm). The significance levels were denoted as follows: ** for $p < 0.01$.

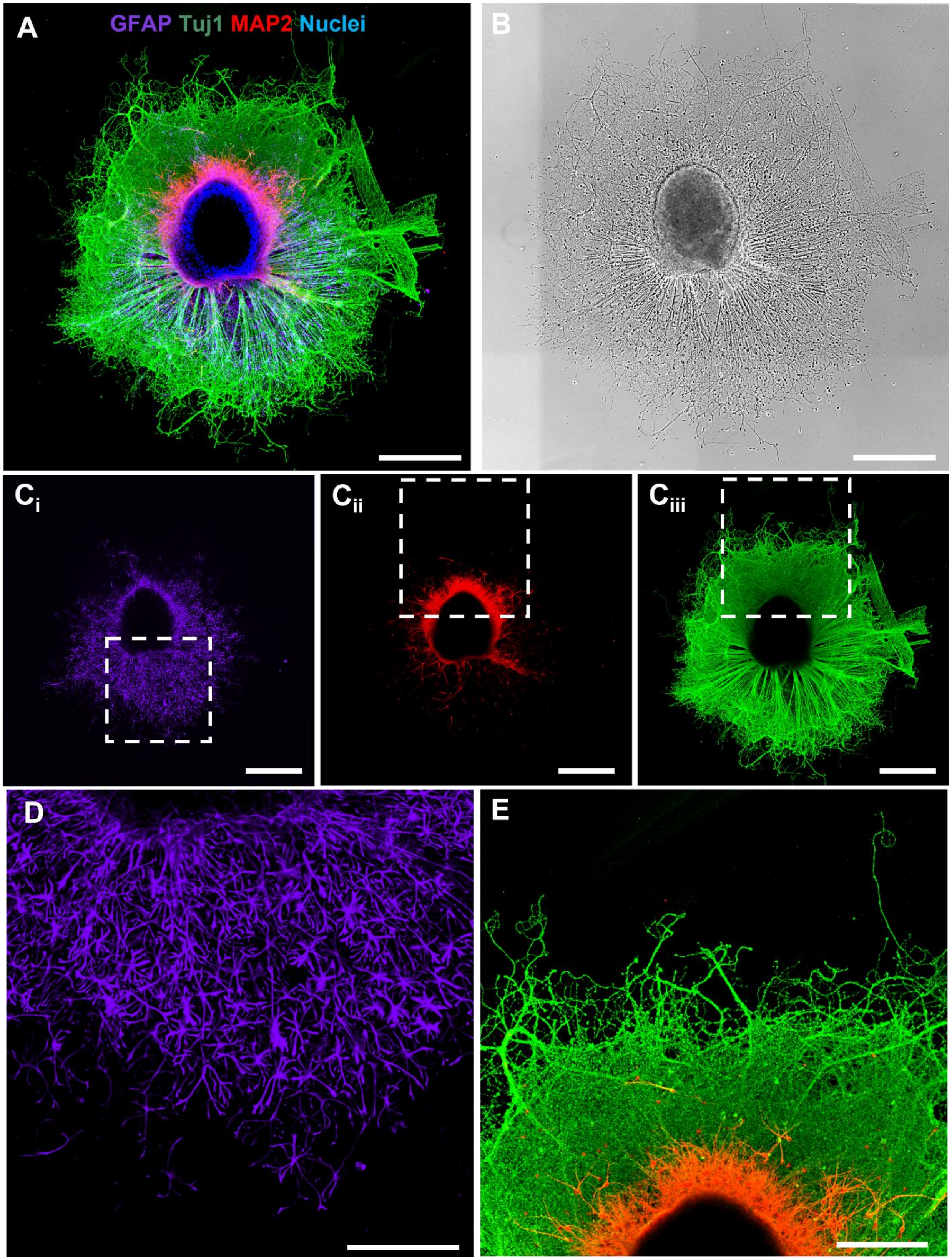


**Supplemental Figure 5**: Qualitative assessment of segmented hippocampal explant at 7 DIV. (A) We utilized Tuj1, MAP2, and GFAP to visualize ($C_i$) astrocytes, ($C_{ii}$) dendrites, and ($C_{iii}$) axons in

hippocampal explants. (B) We took phase images of hippocampal explants to confirm culture viability prior to performing immunocytochemistry. (D) We qualitatively observed preliminary astrocyte migration and quasi-domain formation as early as 7 DIV. (E) We also qualitatively observed that there was no co-localization of MAP2 and Tuj1 signals, which suggests neuronal polarization. (A,B,C: scale Bar = 500 um; D, E: scale Bar = 250 um)
